# Evolution of transcriptional regulation of histidine metabolism in Gram-positive bacteria

**DOI:** 10.1186/s12864-022-08796-y

**Published:** 2022-08-25

**Authors:** German A. Ashniev, Natalia V. Sernova, Aleksei E. Shevkoplias, Ivan D. Rodionov, Irina A. Rodionova, Alexey G. Vitreschak, Mikhail S. Gelfand, Dmitry A. Rodionov

**Affiliations:** 1 A.A. Kharkevich Institute for Information Transmission Problems, RAS, Moscow, Russia; 2 National Research University Higher School of Economics, Moscow, Russia; 3 University of California San Diego, La Jolla, San Diego, CA USA; 4 Skolkovo Institute of Science and Technology, Moscow, Russia; 5 Sanford-Burnham Medical Research Institute, La Jolla, San Diego, CA USA

**Keywords:** Transcription regulation, Comparative genomics, Histidine metabolism, Bacteria, RNA attenuation

## Abstract

**Background:**

The histidine metabolism and transport (*his*) genes are controlled by a variety of RNA-dependent regulatory systems among diverse taxonomic groups of bacteria including T-box riboswitches in Firmicutes and Actinobacteria and RNA attenuators in Proteobacteria. Using a comparative genomic approach, we previously identified a novel DNA-binding transcription factor (named HisR) that controls the histidine metabolism genes in diverse Gram-positive bacteria from the Firmicutes phylum.

**Results:**

Here we report the identification of HisR-binding sites within the regulatory regions of the histidine metabolism and transport genes in 395 genomes representing the Bacilli, Clostridia, Negativicutes, and Tissierellia classes of Firmicutes, as well as in 97 other HisR-encoding genomes from the Actinobacteria, Proteobacteria, and Synergistetes phyla. HisR belongs to the TrpR family of transcription factors, and their predicted DNA binding motifs have a similar 20-bp palindromic structure but distinct lineage-specific consensus sequences. The predicted HisR-binding motif was validated in vitro using DNA binding assays with purified protein from the human gut bacterium *Ruminococcus gnavus*. To fill a knowledge gap in the regulation of histidine metabolism genes in Firmicutes genomes that lack a *hisR* repressor gene, we systematically searched their upstream regions for potential RNA regulatory elements. As result, we identified 158 T-box riboswitches preceding the histidine biosynthesis and/or transport genes in 129 Firmicutes genomes. Finally, novel candidate RNA attenuators were identified upstream of the histidine biosynthesis operons in six species from the *Bacillus cereus* group, as well as in five Eubacteriales and six Erysipelotrichales species.

**Conclusions:**

The obtained distribution of the HisR transcription factor and two RNA-mediated regulatory mechanisms for histidine metabolism genes across over 600 species of Firmicutes is discussed from functional and evolutionary points of view.

**Supplementary Information:**

The online version contains supplementary material available at 10.1186/s12864-022-08796-y.

## Background

**Fig. 1:**
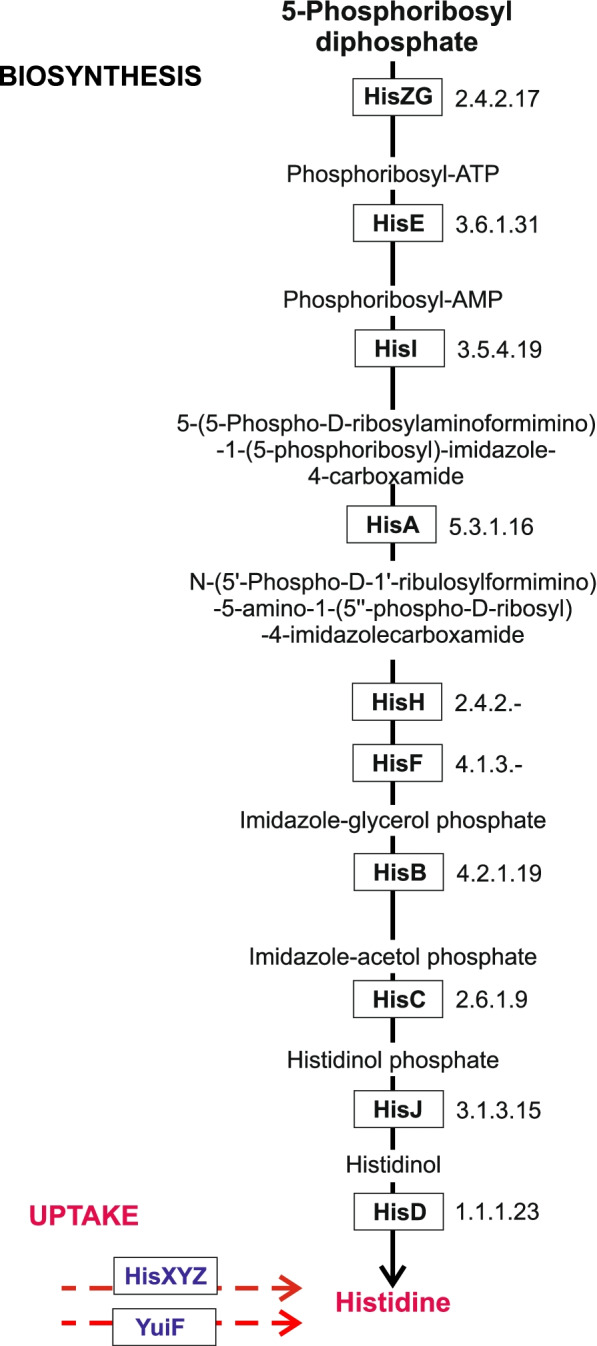
Metabolic pathway of histidine biosynthesis in bacteria

L-histidine (hereafter histidine) is a positively charged proteinogenic amino acid containing an imidazole side chain [1]. Biosynthesis of histidine occurs in most organisms, including bacteria, archaea, some lower eukaryotes, and plants [2, 3]. Among humans, it is considered as an essential amino acid in the diet. Histidine-producing microorganisms (prototrophs) can synthesize this amino acid using a common biosynthetic pathway, which consists of ten biochemical steps (Table 1, Fig. 1) [1, 4]. The histidine biosynthesis genes are organized into compact *his* operons in many Gram-negative and Gram-positive bacteria, although the operon content and gene order are different between the major bacterial phyla. The structure and organization of the *his* operons were extensively studied in Proteobacteria [5], Archaea [6], and other prokaryotes [7]. Broad phylogenetic distribution of the unified *his* operon in bacteria from three major phyla, namely Proteobacteria, Actinobacteria, and Firmicutes, suggested that this operon is indeed ancient, however, a few other lineages of bacteria such as Cyanobacteria possess stand-alone *his* genes that are not clustered into operons [7]. Among Archaea, the *his* genes are scattered on the chromosomes in the majority of Euryarchaeota, in contrast to the Crenarchaeota and Thaumarchaeota, where *his* genes are arranged into the *his* operons of various structures [6]. In the process of evolution, many of the *his* genes underwent fusion, thus affecting the number of genes in the *his* operon [8]. Among eleven individual genes from the histidine biosynthesis pathway, at least seven genes (*hisF-hisI-hisE*, *hisZ-hisG*, *hisJ-hisB*) have undergone various single or multiple fusions in a variety of genomes [8, 9].

**Table 1:** Functional annotations of genes from the histidine metabolism subsystem

Name	Functional role
HisZ	ATP phosphoribosyltransferase (EC 2.4.2.17)
HisG	ATP phosphoribosyltransferase regulatory subunit (EC 2.4.2.17)
HisD	Histidinol dehydrogenase (EC 1.1.1.23)
HisB	Imidazoleglycerol-phosphate dehydratase (EC 4.2.1.19)
HisH	Imidazole glycerol phosphate synthase amidotransferase subunit (EC 2.4.2.-)
HisA	Phosphoribosylformimino-5-aminoimidazole carboxamide ribotide isomerase (EC 5.3.1.16)
HisF	Imidazole glycerol phosphate synthase cyclase subunit (EC 4.1.3.-)
HisI	Phosphoribosyl-AMP cyclohydrolase (EC 3.5.4.19)
HisE	Phosphoribosyl-ATP pyrophosphatase (EC 3.6.1.31)
HisC	Histidinol-phosphate aminotransferase (EC 2.6.1.9)
HisJ	Histidinol-phosphatase (EC 3.1.3.15)
HisR	Histidine repressor, TrpR family
HisX	Histidine ABC transporter, histidine-binding protein
HisY	Histidine ABC transporter, permease protein
HisZ*	Histidine ABC transporter, ATP-binding protein
YuiF	Histidine permease YuiF

The HisZ component of histidine ABC transporter is marked with asterisk to distinguish it from eponymous enzyme

Histidine biosynthesis and transport genes can be regulated by a variety of mechanisms including a repressor protein and various RNA structures, such as transcriptional attenuators and T-box riboswitches. In *E. coli* and other g-proteobacteria, the *his* operon is regulated by an attenuation mechanism, which is based on coupling between transcription and translation of the leader peptide containing tandem histidine codons [10, 11]. Attenuators are upstream regulatory regions that fold into one of two alternative RNA structures. During attenuation, the ribosome becomes stalled in the leader region under the condition of amino acid starvation, when the level of charged tRNA is low, thus promoting the formation of antiterminator structure and transcription readthrough. At high level of histidine, the ribosome proceeds to the end of the leader peptide, promoting the formation of an alternative structure that prematurely terminates the transcription of *his* operon. In some attenuators, the transcriptional terminator is replaced with another regulatory structure that sequesters the ribosomal-binding site (RBS) of the first gene in the regulated operon, thus functioning at the translation, not transcription level [1].

In *Corynebacterium glutamicum* and *Lactococcus lactis*, the *his* operon is regulated via the T-box transcriptional attenuation mechanism [12, 13]. The T-box riboswitches are broadly distributed in Actinobacteria and Firmicutes, where they control the expression of many genes encoding various aminoacyl-tRNA synthetases and selected amino acid biosynthesis enzymes and transporters [14]. T-box regulatory elements are characterized by a set of conserved RNA structures, one of whose containing the specifier sequence, which is complementary to the anticodon sequence of tRNA [15]. This specifier sequence binds the corresponding tRNA, and depending on its charging status either promotes the formation of the transcriptional terminator, or stabilizes an alternative RNA structure (antiterminator). T-box-dependent regulation of the *his* biosynthesis operon and the candidate histidine ABC-transporter operon hisXYZ* was found among the Lactobacillales order, as well as in *Listeria monocytogenes* (Bacillales) and *Clostridioides difficile* (Eubacteriales) [14]. However, the histidine-dependent transcriptional regulation is poorly studied in other Gram-positive bacteria from the Firmicutes phylum. Previously, we have identified a novel potential regulator of the histidine metabolism, named HisR, that is encoded by the hypothetical gene *yerC* in *B. subtilis* and its ortholog in *Staphylococcus aureus* [16, 17]. The inferred HisR regulons include the histidine biosynthesis *his* operon and the putative histidine uptake permease *yuiF*. The predicted histidine repressor HisR belongs to the TrpR family of tryptophan-sensing repressors [16, 17]. A putative TrpR-like repressor was also implicated in the control of the *his* operon in a Gram-negative organism, *Xanthomonas oryzae* [18], however, its regulatory mechanism, DNA-binding sites, and molecular effectors have not been studied.

In this study, we report a detailed analysis of the histidine biosynthesis and uptake genes and their regulation by the HisR regulator and RNA regulatory mechanisms in many available genomes of Firmicutes, as well as other taxonomic groups of bacteria possessing *hisR* orthologs. We identified DNA binding sites of HisR regulators and reconstructed their regulons in the majority of Firmicutes and other taxonomic lineages. To evaluate the accuracy of the genomic reconstructions of the HisR regulon, we performed experimental testing of the predicted regulatory site upstream of the *his* operon in *Ruminococcus gnavus* using in vitro binding assay with purified HisR protein. As result, we confirmed that HisR binds to its DNA binding site, and that L-histidine is required for HisR-DNA binding. Comparison of HisR proteins and their predicted DNA motifs with that of the previously characterized tryptophan repressor TrpR in *E. coli* provided insights into the mechanism of HisR action. Analysis of the distribution of RNA regulatory elements for histidine metabolism genes in taxonomic lineages without HisR allowed us to propose a likely evolutionary scenario for histidine regulons of Firmicutes.

## Results and discussion

### Phylogenetic distribution of *hisR* genes

We selected a set of 626 representative genomes of Firmicutes and classified them into 11 taxonomic groups, with three major groups being the Bacillales, Lactobacillales, and Eubacteriales orders. Using this genome set, we identified orthologs of the HisR regulator from *Bacillus subtilis* (encoded by the *yerC* gene) [11]. Orthologs of HisR were identified in 395 Firmicutes genomes, as well in 97 bacterial genomes from other phyla (Additional file 1). Among 137 genomes from the Bacillales order, HisR was identified in all genomes except the *Bacillus cereus* group (six closely related species), *Gemella* spp., and *Macrococcus caseolyticus*. Among the Eubacteriales order (195 genomes) HisR is absent from 21 genomes, most of which belong to the Peptostreptococcaceae family, e.g. *Clostridioides difficile* (Additional file 2). HisR orthologs were identified among all analyzed genomes from the following orders: Acidaminococcales (4 genomes), Halanaerobiales (7 genomes), Natranaerobiales (1 genome), Selenomonadales (16 genomes), and Veillonellales (11 genomes). Among the Thermoanaerobacterales (43 genomes) and Tissierellales (18 genomes) orders, HisR is missing in seven and two genomes, respectively. In contrast, HisR orthologs are absent in the majority of 174 analyzed Lactobacillales genomes, being only found in three *Enterococcus* spp. and *Carnobacterium maltaromaticum* (44% and 53% identity, respectively, as compared to HisR from *B. subtilis*). As discussed below, the reconstruction of regulons for these rare members of the HisR regulator family in Lactobacillales suggests that their function is unrelated to the histidine biosynthesis or uptake. Finally, all 20 genomes from the Erysipelotrichales order of Firmicutes lack HisR orthologs. Thus, although HisR regulator is broadly distributed among Firmicutes, it was apparently lost in several independent taxonomic groups and in multiple individual species. It should be noted that among 231 genomes that lack a *hisR* regulator, there are both histidine auxotrophs missing histidine biosynthesis and prototrophs that possess the *his* genes. In this study, we analyzed the HisR binding sites and reconstructed corresponding regulons in the genomes possessing this transcription factor and also identified RNA regulatory mechanisms for *his* genes in the majority of remaining genomes that lack HisR orthologs.

**Fig. 2:**
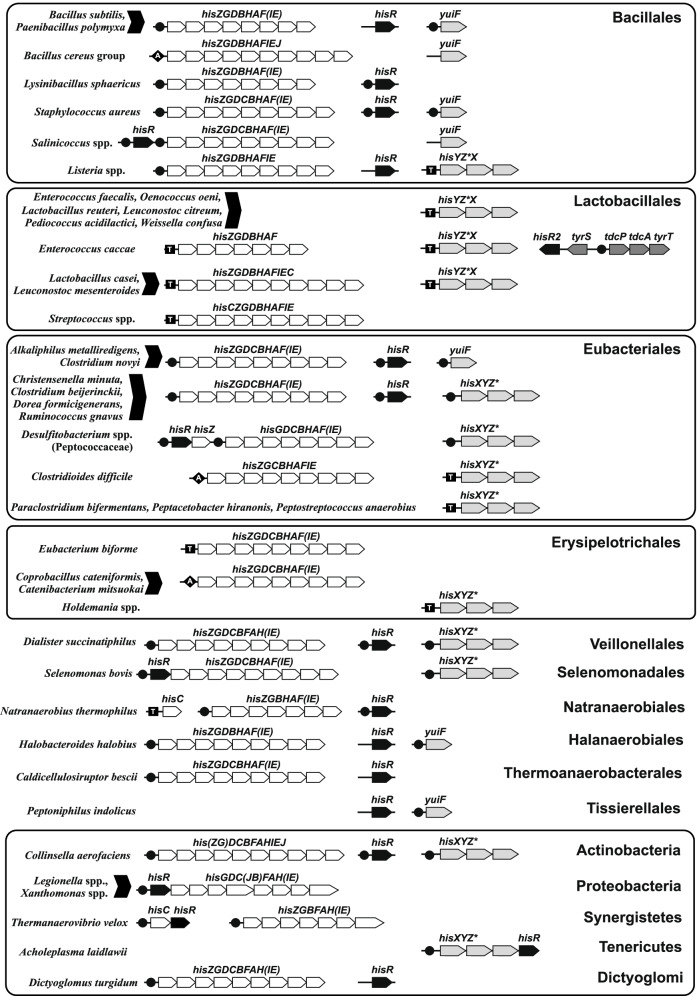
Genomic context of the reconstructed HisR and RNA regulons for histidine metabolism genes in Firmicutes and other bacterial phyla. Histidine biosynthesis and transport genes are shown by white and gray arrows, respectively. HisR repressor genes are in black. Candidate HisR-binding sites are shown by black circles. T-boxes and RNA attenuators are shown by black squares and rhombuses with ‘T’ and ‘A’ letters inside. Content of the reconstructed HisR regulons in other species and candidate HisR binding sites are listed in Additional files 3 and 4

Co-localization of a transcription factor (TF) gene with its cognate regulated genes is common for local TF regulons involved in regulation of specific metabolic pathways in bacteria [19–21]. We analyzed the genomic neighborhoods of the identified *hisR* genes in Firmicutes and specifically checked for cases of co-localization of *hisR* with genes involved in histidine biosynthesis or transport (Fig. 2 and Additional file 2). In most of the analyzed Firmicutes genomes, *hisR* is not chromosomally clustered with *his* genes. However, *hisR* was found immediately upstream of the histidine biosynthesis operon in all twenty analyzed genomes from the Peptococcaceae family, in most genomes from the Selenomonadales and Acidaminococcales orders of Negativicutes (18 out of 20 genomes), in six genomes from the Syntrophomonadaceae and Thermoanaerobacteraceae families, as well as in two *Salinicoccus* species (the Bacillales order).

In addition to Firmicutes, HisR orthologs were identified in a small subset of genomes representing other bacterial phyla (Additional file 3). Among g-proteobacteria, HisR was found in 51 genomes including many human pathogens (*Legionella*, *Francisella*, and *Wohlfahrtiimonas*) and plant pathogens from the Xanthomonadales order. HisR was also found in 11 genomes from the Rhodobacterales and Caulobacterales orders of a-proteobacteria, many of which were isolated from marine or extremely cold environments. In the majority of Proteobacteria, *hisR* is the first gene of *his* operon, suggesting its local character of regulation. Additional *hisR* orthologs were found in 22 genomes of Actinobacteria from the orders of Coriobacteriales and Eggerthellales, where they were never found in chromosomal clusters with the histidine biosynthesis and transport genes. In six out of nine genomes from the Synergistetes phylum, *hisR* forms a putative operon with *hisC*, while in *Pyramidobacter piscolens* it belongs to the *his* operon. Finally, a *hisR* ortholog was identified in a single species of Mollicutes, *Acholeplasma laidlawii* (forming an operon with the hisXYZ* transporter), two *Dictyoglomus* species from the phylum of Dictyoglomi (as a standalone gene), and *Elusimicrobium minutum* from the phylum of Elusimicrobia (as the first gene of the *his* operon).

Similar to other bacterial TFs controlling the central metabolic pathways, such as ArgR, TrpR, MetJ, and TyrR repressors in g-proteobacteria [22], HisR regulators are present in a single copy in most genomes with a notable exception of four Synergistetes, five Eubacteriales, and two Bacillales genomes possessing two *hisR* paralogs (Additional files 2 and 3). To get insight into the possible evolutionary history of the duplicates, we constructed the phylogenetic tree of all HisR proteins identified in a representative subset of analyzed genomes (Additional file 1). One *hisR* paralog in *Paenibacillus alvei* is located in a conserved gene cluster among most *Paenibacillus* spp., while a *hisR2* paralog is conservatively located within another gene cluster, *tdcP-tdcA-tyrT*, encoding a tyrosine/tyramine exchanger, tyrosine decarboxylase, and a putative tyrosine transporter [23]. Chromosomal co-localization of the *hisR2* and *tdcP-tdcA-tyrT* genes was also identified in histidine auxotrophic bacterium *Carnobacterium maltaromaticum*. Furthermore, only three species of *Enterococcus* possess a rare gene cluster containing *hisR2*, *tdcP-tdcA-tyrT* and the tyrosyl-tRNA synthetase gene *tyrS* (Fig. 2). Identification of candidate HisR2 binding sites upstream of the *tdcP* genes (see below) suggests that these HisR2 paralogs could have acquired a new function in the control of tyrosine/tyramine metabolism. Finally, one of two *hisR* paralogs in *Oscillibacter* spp. is located within a conserved gene cluster with histidine utilization genes, namely histidine ammonia-lyase *hutH* and histidine permease *hisT*, suggesting another potential case of functional specialization of HisR regulators.

### Identification of HisR binding motifs and regulons

**Fig. 3:**
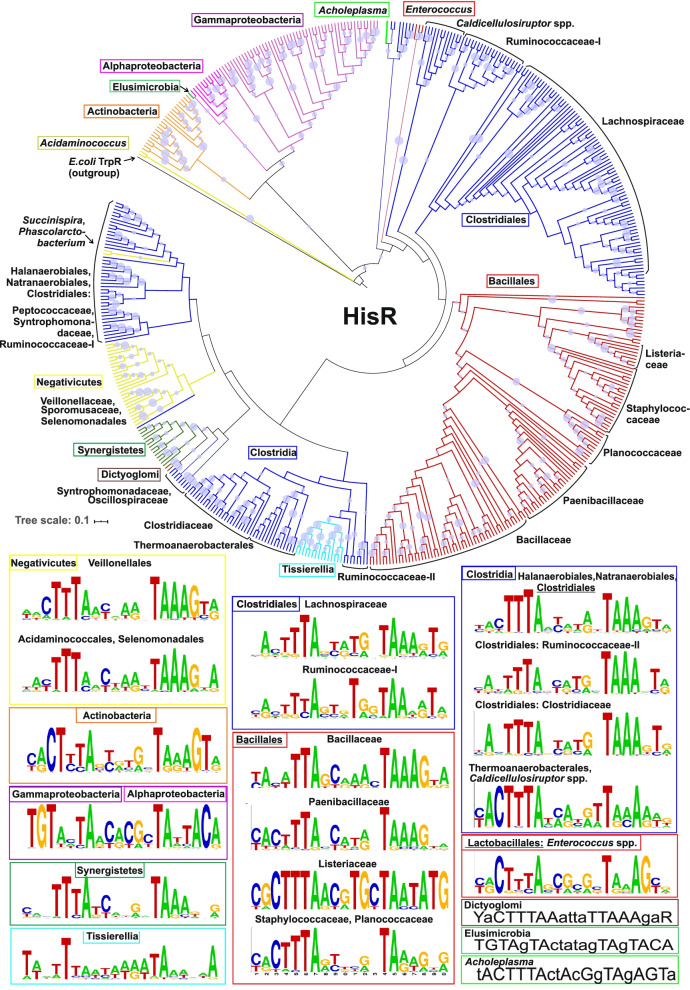
DNA binding motifs and phylogeny of HisR regulators in various taxonomic groups of Bacteria. Sequence logos representing the consensus HisR binding site motifs were constructed using all candidate sites in the respective bacterial lineage. Maximum likelihood phylogenetic tree for HisR proteins in studied genomes of Firmicutes (395 genomes), Proteobacteria (62 genomes), Actinobacteria (22 genomes), Synergistetes (9 genomes), Dictyoglomi (2 genomes), Elusimicrobia and Tenericutes. Branches representing HisR proteins from each of the above phyla are highlighted by colors matching their corresponding HisR motif box outline colors. The size of circles in internal nodes represents bootstrap values in percentages (out of 100). TrpR repressor from *E. coli* was used as an outgroup. Detailed tree with protein IDs and genome names is presented in the rectangular form in Additional file 1

To reconstruct HisR regulons, we applied the comparative genomics approach [24] that combines the search for candidate regulator-binding sites with the cross-genomic comparison of regulons. To facilitate the comparative genomics analysis, all analyzed genomes of Firmicutes containing HisR regulators were subdivided into individual taxonomic groups, namely Bacillaceae, Listeriaceae, Paenibacillaceae, Staphylococcaceae, Eubacteriales, Halanaerobiales, Thermoanaerobacterales, Acidaminococcales/Selenomonadales, Veillonellales, and Tissierellales. A search for palindromic motifs was performed within putative promoter regions between –350 nt upstream and 50 nt downstream to the translational start of genes involved in the histidine biosynthesis and uptake (Additional file 2). As result, in each taxonomic group, we identified the lineage-specific HisR binding DNA motifs and constructed the corresponding Positional Weight Matrices (PWMs) used to search for additional candidate HisR sites in the upstream regions of histidine metabolisms genes and *hisR* repressors (Additional file 4). Weak sites with scores below the threshold were further validated via phylogenetic footprinting by constructing multiple alignments of orthologous upstream gene regions (see Methods). As the result, we confirmed 40 weak HisR sites, mostly from Eubacteriales genomes (Additional file 5). Also, we applied this approach to HisR-containing genomes from three other phyla, namely Actinobacteria, Proteobacteria, and Synergistetes (Additional file 3). The obtained candidate HisR operator sites share a similar 20 bp palindromic structure, which corresponds to the structure of the DNA motif of homologous TrpR repressors from g-proteobacteria [23]. Sequence logos for HisR-binding DNA motifs were drawn with WebLogo using all identified candidate sites in the genomes from the same lineage (Fig. 3). Interestingly, the majority of identified lineage-specific HisR motifs in Firmicutes, Actinobacteria, and Synergistetes have a common consensus (cacTTTAnnnnnnTAAAgtg), which is different from the TrpR binding motif (TGTAcTnGTnnACnAgTACA). In contrast, the predicted HisR motif in g- and a-proteobacteria (TGTAnTArnnnnyTnTTACA) is quite similar to the TrpR motif. The latter observation is in agreement with the phylogenetic tree of HisR and TrpR proteins, which features the proteobacterial branch of HisR proteins most closely related to TrpR.

The content of reconstructed HisR regulons in Firmicutes is quite uniform in most analyzed lineages, as it includes the histidine biosynthesis genes (often organized into a single *his* operon) and/or standalone genes/operons encoding histidine transporters from two major families (Fig. 2, Table 2). The ABC-family transporter HisXYZ* is controlled by HisR in bacteria from the Clostridia and Negativicutes classes, whereas the YuiF permease is a common member of HisR regulons in the Bacillales order. Among Bacillales, additional HisR-binding sites were identified upstream of *hisR* gene in a subset of genomes including *Staphylococcus*, *Salinicoccus*, *Kurthia*, and *Ureibacillus* spp., suggesting an autoregulation mechanism. Noteworthy, autoregulation is common among other amino acid-specific repressors including ArgR, MetJ, TrpR, and TyrR in g-proteobacteria [22].

**Table 2:** Distribution and functional content of HisR regulons in Firmicutes

Order	Genera (examples)	Target genes	Count
**Bacillales** (126 genomes)	*Bacillus; Brevibacillus; Geobacillus; Paenibacillus;* etc	*his* operon	40
	*Bacillus; Brevibacillus; Paenibacillus; Solibacillus; Staphylococcus* etc	*his* operon; *yuiF*	52
	*Desmospora; Kurthia; Lysinibacillus; Salinicoccus;* etc	*his* operon; *hisR*	26
**Lactobacillales** (4 genomes)	*Enterococcus, Carnobacterium*	*tdcP-tdcA-tyrT*	4
**Eubacteriales** (169 genomes)	*Clostridium; Eubacterium; Ruminococcus; Subdoligranulum;* etc	*his* operon	22
	*Alkaliphilus; Clostridium*	*his* operon; *yuiF*	2
	*Christensenella; Anaerostipes; Blautia; Butyrivibrio; Coprococcus;* etc	*his* operon; *hisR*	114
	*Lachnobacterium; Lachnoclostridium; Bryantella; Roseburia;* etc	*his* operon; hisXYZ*	86
	*Clostridium*	*yuiF*	3
	*Clostridium; Eubacterium; Caloramator*	hisXYZ*	6
	*Eubacterium; Blautia; Oribacterium; Peptococcus; Flavonifractor;* etc	*hisR*; hisXYZ*	8
**Halanaerobiales** (7 genomes)	*Halanaerobium; Halothermothrix*	*his* operon	2
	*Halanaerobium; Halobacteroides*	*his* operon; *yuiF*	3
	*Acetohalobium*	*his* operon; *hisR*	1
	*Orenia*	*his* operon; hisXYZ*	1
**Natranaerobiales**	Natranaerobius (1 genome)	*his* operon; *hisR*	1
**Thermoanaero-bacterales** (36 genomes)	*Caldicellulosiruptor; Desulfovirgula; Moorella; Caldanaerovirga;* etc	*his* operon	29
	*Carboxydothermus; Thermoanaerobacter*	*his* operon; *hisR*	7
**Acidaminococcales** (4 genomes)	*Acidaminococcus*	*his* operon; *hisR*	2
	*Phascolarctobacterium; Succinispira*	*his* operon; hisXYZ*	2
**Selenomonadales** (16 genomes)	*Megamonas; Mitsuokella;Selenomonas; Anaeromusa; Thermosinus*	*his* operon; hisXYZ*; *hisR*	14
	*Pelosinus; Selenomonas*	hisXYZ*; *hisR*	2
**Veillonellales** (11 genomes)	*Dialister; Megasphaera; Veillonella*	*his* operon; hisXYZ*; *hisR*	8
	*Anaeroglobus; Megasphaera; Veillonella*	*his* operon; hisXYZ*	7
**Tissierellales** (13 genomes)	*Anaerococcus; Finegoldia; Parvimonas*	*hisR*	7
	*Gottschalkia*	*his* operon	1
	*Peptoniphilus*	*yuiF*	5
**TOTAL number of operons controlled by HisR (387 genomes):**			**635**

The HisZ component of histidine ABC transporter is marked with asterisk to distinguish it from eponymous enzyme

In Proteobacteria, HisR almost exclusively controls the *his* operon, which includes *hisR* as the first gene, and the predicted HisR binding site positioned immediately upstream of *hisR* (Fig. 2). In a single species of *Wohlfahrtiimonas chitiniclastica*, the HisR regulon includes both the *his* operon and the hisR-hisXYZ* operon encoding histidine transporter. In 20 genomes of Actinobacteria, HisR controls three standalone gene loci — the *his* operon, hisXYZ* transporter, and *hisR*. In contrast, the HisR regulon in two histidine auxotrophic species of Actinobacteria, *Collinsella intestinalis* and *Cryptobacterium curtum,* contains only the hisXYZ* and *hisR* genes. Among Synergistes, HisR controls the *his* operon and the *hisC-hisR* operon in eight genomes. A single HisR-binding site was identified upstream of the *hisR-his* operon in the genomes of *Pyramidobacter piscolens* (Synergistes) and *Elusimicrobium minutum* (Elusimicrobia). In both *Dictyoglomus* species, a HisR-binding site was identified upstream the *his* operon, whereas *hisR* is not autoregulated. *Acholeplasma laidlawii* is the only *hisR*-containing species among the Mollicutes phylum. The genome of this histidine auxotrophic bacterium contains a single HisR-binding site located upstream of the hisXYZ*-hisR operon.

### T-box mediated regulation of histidine metabolism in Firmicutes

The T-box leader is a highly conserved RNA regulatory element that is involved in regulation of various genes involved in amino acid metabolism in Gram-positive bacteria, mostly in the Firmicutes phylum [25]. T-box elements initially identified in the regulatory leader regions of aminoacyl-tRNA synthetase genes and some amino acid biosynthetic genes in *Bacillus subtilis* and related bacteria control gene expression via a unique transcription antitermination mechanism. T-box serves as a riboswitch that binds directly to a specific uncharged tRNA and thus measures the amino acid availability in the cell.

We searched the upstream regions of histidine biosynthesis and transport genes from Firmicutes genomes to identify T-box RNA regulatory elements described by the RF00230 family in the Rfam database [26]. As a result, we identified candidate T-boxes motifs upstream of 159 genes/operons involved in histidine metabolism in 129 out of 395 studied genomes of Firmicutes (Table 3). The largest number of T-box regulatory elements (85%) was identified in the Lactobacillales order, where they replace the missing HisR repressor. Most of these genomes contain a single T-box in each regulatory region, however a few cases of tandem riboswitches were identified upstream of the histidine transporter operon in five *Enterococcus* species (Additional file 4). Double T-boxes have been previously reported for a number of amino acid metabolism genes/operons in diverse species of Firmicutes, where they were potentially evolved by recent duplication events and might function cooperatively leading to a sharper response to uncharged tRNA concentrations [14].

**Table 3:** Distribution of RNA regulatory elements for histidine metabolism in Firmicutes

Order	Genera	Target genes	Count
**T-boxes** (129 genomes)**:**			
**Bacillales**	*Listeria*	hisYZ*X	6
**Lactobacillales** (106 genomes)	*Lactobacillus, Lactococcus, Streptococcus, Enterococcus, Leuconostoc, Oenococcus, Aerococcus*	*his* operon	56
	*Lactobacillus, Enterococcus, Leuconostoc, Oenococcus, Pediococcus, Weissella, Melissococcus*	hisYZ*X	77
	*Alloiococcus, Dolosigranulum*	*yuiF*	2
**Eubacteriales**	*Alkaliphilus, Clostridioides, Filifactor, Paraclostridium Peptostreptococcus, Peptacetobacter*	hisXYZ*	7
**Erysipelotrichales** (9 genomes)	*Eubacterium, Allobaculum, S. pleomorphus, C. innoculum*	*his* operon	7
	*Holdemania, Clostridium innoculum*	hisXYZ*	3
**Natranaerobiales**	Natranaerobius	*hisC*	1
**TOTAL number of operons controlled by T-box(His):**			**159**
**RNA attenuators** (17 genomes)**:**			
**Bacillales**	*Bacillus cereus* group	*his* operon	6
**Eubacteriales**	*Lutispora;* Peptostreptococcaceae: *Acetoanaerobium, Clostridioides, Intestinibacter, Terrisporobacter*	*his* operon	5
**Erysipelotrichales**	*Eggerthia, Erysipelatoclostridium, Catenibacterium, Coprobacillus, [Clostridium]*	*his* operon	6
**TOTAL number of operons controlled by T-box(His):**			**17**

The HisZ component of histidine ABC transporter is marked with asterisk to distinguish it from eponymous enzyme

Within the Lactobacillales lineage, T-boxes were found in upstreams regions of histidine biosynthesis and/or transport operons (Fig. 2). For example, *Leuconostoc* and many histidine prototrophic *Lactobacillus* species possess two His-specific T-boxes preceding the *his* biosynthetic and hisYZ*X transport operons. Among the *Streptococcus* genus, T-boxes were found only upstream of the *his* operons, whereas the hisYZ*X operons are not regulated by any known mechanism. Among the *Enterococcus* genus, there are both histidine prototrophs with T-box-dependent regulation of *his* operons and histidine auxotrophs that have a hisYZ*X transporter under T-box regulation. However, a subset of Lactobacillales genomes have no recognized regulation of the genes of interest. Interestingly, two auxotrophic members of the Lactobacillales order, *Alloiococcus otitis* and *Dolosigranulum pigrum*, possess a T-box-regulated histidine transporter *yuiF*, which is commonly regulated by HisR in the Bacillales order. Besides Lactobacillalles, the *Listeria* genus (class Bacillales) also developed the T-box regulation. Interestingly, both HisR and T-box regulatory mechanisms are combined in *Listeria* spp., where the *his* operon is under HisR control, whereas the hisYZ*X transporter is regulated by His-specific T-box. This configuration can be explained by possible horizontal transfer of the T-box-regulated hisYZ*X transporter from the Lactobacillales order. Indeed, each of the three components of this ABC transporter from *Listeria* spp. clusters with their respective orthologs from *Lactobacillus* spp. demonstrating 55–60% amino acid identity.

Similar to Bacillales, the Eubacteriales order demonstrates a very rare occurrence of T-boxes upstream of histidine metabolism genes, which correlates with a wide distribution of HisR-dependent regulation. Among the Clostridiaceae family, a histidine-specific T-box was only found upstream of the hisXYZ* transporter in *Alkaliphilus oremlandii*, which lacks HisR regulator. The closest hits for the components of this T-box-regulated transporter belong to the HisR-regulated transporter operon from *Clostridium perfringens* (60–65% identity), suggesting that the replacement of its regulatory region by a T-box leader is the most plausible evolutionary scenario. Among the Peptostreptococcaceae family, T-boxes exclusively control the hisXYZ* transporter operon, suggesting that histidine-specific T-box riboswitches have been acquired in the common ancestor of these species. It should be noted that the *his* biosynthetic operons that are present in some Peptostreptococcaceae genomes are regulated by a different RNA-based mechanism, attenuation (see below). In the Natranaerobiales class, there is only one genome in the reference set, *Natranaerobius thermophilus*, which has a T-box upstream of a single gene *hisC*, whereas other histidine biosynthesis genes are controlled by the HisR repressor (Fig. 2). Finally, T-boxes were identified upstream of the *his* operon in seven out of thirteen genomes of histidine prototrophic bacteria from the Erysipelotrichaceae family (the Erysipelotrichia class). Additional T-box regulatory elements were identified upstream of the hisXYZ* transporter in two histidine auxotrophic *Holdemania* species, as well as in *Clostridium innocuum*, which has two T-box-regulated operons (Fig. 2). Alternative regulatory mechanisms identified for the *his* operons in other Erysipelotrichaceae species that lack T-boxes are described in the following section.

### Attenuation mechanisms for histidine metabolism genes

The histidine operon leader is an RNA element that has been found upstream of histidine biosynthesis (*his*) operons in *E. coli* and related g-proteobacteria and is involved in the amino acid-dependent attenuation of their transcription [11]. The leader sequence can assume two different secondary structures known as the terminator and the anti-terminator structure. The leader also codes for a very short peptide sequence that is rich in histidine. The terminator structure is recognized as a termination signal for RNA polymerase and the operon is not transcribed. This structure forms when the cell contains an excess of histidine and the ribosome movement along the leader transcript is not impeded. We searched the upstream regions of the histidine metabolism genes in Firmicutes with the proteobacterial-type His-leader motif (RF00514 in the Rfam database) but have not identified any occurrence of this RNA motif in this bacterial phylum.

We further checked the upstream regions of *his* operons without HisR- or T-box-mediated regulation for the presence of a potential histidine-rich leader peptide. As result, we identified three lineages of Firmicutes that may rely on transcriptional attenuation mechanisms to regulate histidine-related genes, including (i) a group of six closely related *Bacillus* species from the *B. cereus* group; (ii) a group of four related species from the Peptostreptococcaceae family (e.g., *C. difficile*) and *Lutispora thermophila* from the Eubacteriales order; and (iii) six different species from the Erysipelotrichales order (Table 3, Fig. 2). We further generated multiple alignments of these upstream regions in each of these taxonomic groups and identified conserved elements of RNA attenuators (Additional file 6), specifically, upstream leader peptides and downstream stretches of complementary RNA nucleotides forming potential attenuator and terminator hairpins, as well as alternative antiterminator structures that are characteristic of the attenuation mechanism. The presumed short leader peptides containing three to five histidine codons in a row were located immediately upstream of candidate attenuator structures.

### Structural analysis of HisR proteins

**Fig. 4:**
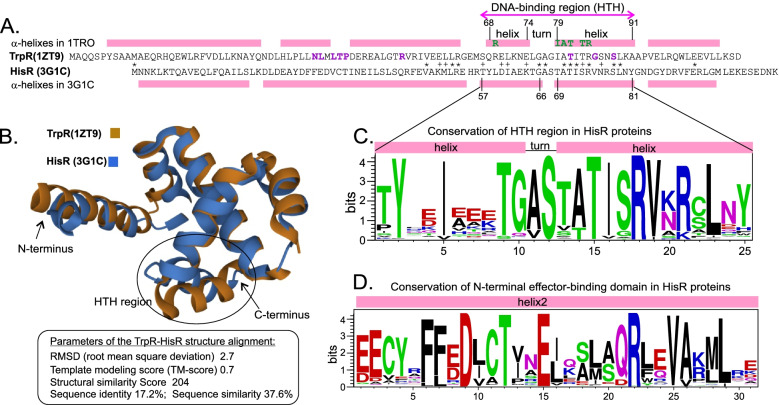
Structural analysis of HisR and TrpR regulators. **A** Alignment of TrpR repressor from *E. coli* and HisR protein from *Eubacterium eligens*. An asterisk marks identical residues. Pink boxes mark the position of alpha-helices in both structures. Helix-turn-helix (HTH) motif of DNA binding region is marked. Residues involved in contacts between TrpR and DNA operator within the HTH motif are shown in green (according to [28]). Tryptophan binding residues in TrpR are shown in purple (according to the 1ZT9 structure). **B** Pairwise structure alignment of *E. coli* TrpR (1ZT9) and *E.eligens* HisR (3G1C) obtained by the RCSB PDB web tool. **C** Sequence logo of the HTH motif in HisR proteins. Logo was built based on alignment of all analyzed HisR proteins from 492 genomes of Firmicutes and other lineages. Positively and negatively charged amino acids are shown in blue and red, respectively

Novel histidine-related transcriptional regulators HisR belong to the TrpR family of tryptophan-sensing repressors (PF01371 in Pfam database), which is a part of a larger clan (CL0123) of DNA-binding proteins containing the helix-turn-helix (HTH) domain. HisR and TrpR proteins are characterized by relatively short lengths (< 100 a.a.) and low pairwise sequence similarity. For example, a BLAST-generated alignment of the TrpR protein from *E. coli* and HisR from *Eubacterium eligens* shows a low but significant similarity between their DNA-binding domains with score = 20, expect = 0.002, identity = 34%, and coverage = 30% (Fig. 4A). We further compared the solved tertiary structures of *E.eligens* HisR protein (PDB ID code 3G1C) and *E. coli* TrpR holorepressor in complex with tryptophan (PDB ID code 1ZT9). Pairwise structure alignment confirmed similar positioning of a-helixes in these proteins and overall structural similarity with root mean square deviation (rmsd) between the aligned pairs of the backbone C_a_ atoms of 2.7 Å (Fig. 4B). The DNA-binding HTH domains of HisR/TrpR consist of two helices connected by a flexible loop. Based on structural analyses of the TrpR-DNA complex, the previously proposed DNA-binding mechanism involves tryptophan-dependent binding of a TrpR monomer to the consensus half-site GNACT and eight base-pair spacer between two half-sites [27]. TrpR interacts with DNA primarily through residues located within the second helix [28], and these DNA-interacting residues are mostly conserved in HisR (Fig. 4A), suggesting both proteins rely on similar mechanisms for DNA recognition. In contrast, among nine tryptophan-contacting residues in TrpR only two amino acids are conserved in HisR and these are located within the second helix of the HTH motif.

To assess the conservation of key structural features in HisR proteins from diverse taxonomic lineages we analyzed multiple alignment of ~ 500 proteins that were previously used for the HisR phylogenetic tree construction (Additional file 1). The DNA-binding domain of HisR proteins contains highly conserved residues within the second helix and the ‘turn’ region, as well as the first two and the last two residues within the first helix of the HTH motif (see sequence logo in Fig. 4C). Thus, three out of six DNA-contacting residues in TrpR (Ala80, Thr 81, Arg84) are conserved in most HisR proteins, while Arg69, Ile79, and Thr83 are replaced with highly conserved Tyr, and less conserved Thr and Ser, respectively. The long helix 2 in HisR is the most conserved region in HisR proteins (Fig. 4D). However, the tryptophan-contacting residues in the corresponding helices 3 and 4 in TrpR are all replaced with different amino acids that are highly conserved among HisR proteins. This suggests that histidine, which has been confirmed to play a role as a co-repressor in HisR (see the experimental validation section below), is recognized by a completely different arrangement of amino acids within helix 2 in comparison with tryptophan binding in TrpR. Of note, among the helix 2 amino acid positions that are highly conserved in the HisR family, there are five Glu and one Asp residues. These six negatively charged residues might be potentially involved in the interaction with the positively charged histidine; however, the exact mechanism of effector recognition by HisR proteins requires further experimental work, e.g. obtaining a crystal structure of the HisR holorepressor in complex with histidine.

### Plausible evolutionary scenarios for regulation of histidine metabolism in Firmicutes

**Fig. 5:**
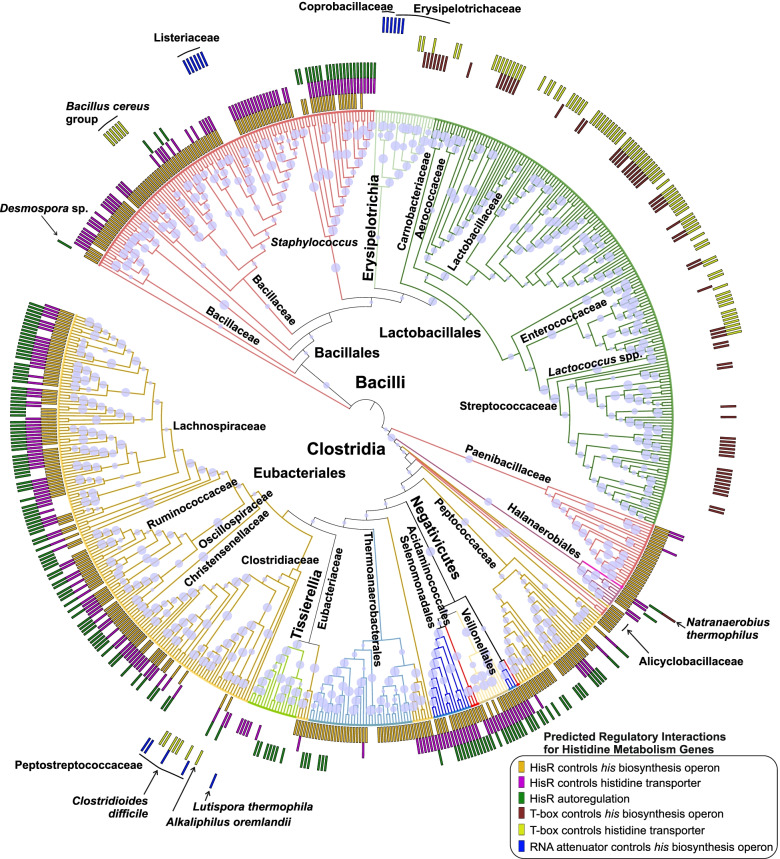
Distribution of various regulatory mechanisms for histidine metabolism genes among analyzed Firmicutes species. The maximum likelihood phylogenetic tree of Firmicutes species was constructed by MEGA X based on concatenated sequences of ribosomal proteins from the analyzed genomes. Colored bars show the occurrence of specific regulatory interactions in each species according to legend and Additional file 2. The size of circles in internal nodes represents bootstrap values in percentages (out of 100). Detailed tree with full genome names is presented in the rectangular form in Additional file 7

Histidine biosynthesis and transport genes in Firmicutes are regulated by different mechanisms in various species. To study the evolution of the identified histidine metabolism regulatory systems we constructed the maximum likelihood phylogenetic tree of 626 Firmicutes species analyzed in this work (Additional file 7). The species tree was based on concatenated sequences of ribosomal proteins extracted from the analyzed genomes (see Methods). The distribution of various regulatory interactions identified for histidine metabolism genes and involving three regulatory mechanisms (HisR, T-box, and RNA-attenuator) was visualized on the species tree (Fig. 5). Compared to histidine-specific RNA regulatory elements, the HisR repressor is the most common regulatory mechanism in Firmicutes, which was also identified in a few other bacterial phyla. The majority of regulatory interactions involving HisR repressors include histidine biosynthesis genes (335 genomes), while the *yuiF* and hisXYZ* transporters are regulated by HisR in 85 and 131 genomes, respectively; finally, HisR autoregulates its gene in 183 genomes. Thus, HisR was likely the ancestral regulatory mechanism for the histidine metabolism genes in Firmicutes. The RNA-dependent regulatory mechanisms for histidine metabolism genes are much less abundant and demonstrate a mosaic, lineage-specific pattern of occurrence, suggesting they were introduced later in the evolution of the corresponding lineages and individual species of Firmicutes.

Hence, the most parsimonious evolutionary scenario for regulation of histidine metabolism in Firmicutes is the following. HisR was present in the last common ancestor of all Firmicutes, and it was lost in the Lactobacillales, and Erysipelotrichales orders, as well as several narrow taxonomic groups from the Bacillales and Eubacteriales orders (Fig. 5). In Lactobacillales, the role of HisR in the regulation of histidine metabolism was assumed by the expanded histidine-specific T-box regulon, which initially included only aminoacyl-tRNA synthetases [14]. At that, the T-boxes in Lactobacillales largely control the histidine transporter operon (in 77 out of 108 genomes) and less frequently the *his* biosynthetic operon (57 out of 108 genomes). Bacteria from two distinct lineages lacking a HisR regulon, the Erysipelotrichaceae and Peptostreptococcaceae families, have acquired (or evolved) two different RNA regulatory motifs, either T-box (in 15 genomes) and/or a novel potential RNA attenuator (in 10 genomes) that control the *his* operon and/or hisZ*YX transporter. Finally, the *Bacillus cereus* group of the Bacillales order has independently acquired a novel RNA attenuator controlling the *his* operon.

Some other potential evolutionary events can be deduced from our analysis. The Listeriaceae genomes have acquired the T-box-regulated hisYZ*X operon, potentially from an unknown *Lactobacillus* spp. Another potential horizontal transfer of a target operon together with its RNA control elements is the *his* operon preceded by an RNA attenuator in *Lutispora thermophila* (a bacterium from the Clostridiaceae family), which is the best hit to the RNA attenuator-controlled *his* operons from *C. difficile* and other Peptostreptococcaceae genomes. In a few cases, a target operon, which is commonly regulated by HisR among the Eubacteriales genomes, has obtained RNA-dependent regulation in a single genome (as compared to closely-related species), such as the acquisition of a T-box upstream element by the hisXYZ* operon in *Alkaliphilus oremlandii* and the *hisC* gene in *Natranaerobius thermophilus*. Thus, the latter bacterium from the Natranerobiales order is another example (in addition to *Listeria* spp.) that implicates both HisR and T-box in the regulation of the histidine metabolism.

### Experimental validation of the HisR regulon

**Fig. 6:**
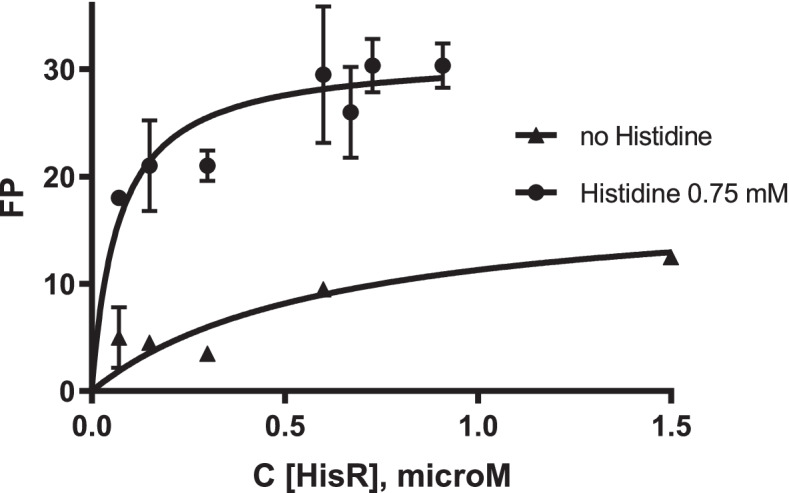
DNA binding assay of HisR regulator from *Ruminococcus gnavus*. Fluorescence polarization binding assay of HisR protein to cognate DNA site upstream of the *hisX* gene in *R. gnavus* and the influence of L-histidine on protein-DNA binding. DNA–protein complex formation was monitored by an increase in the fluorescence polarization (FP) value. The error bars indicate standard deviations of triplicate values. Increasing concentrations of HisR protein were mixed with a 30-bp fluorescence-labeled DNA fragment of the *hisX* gene promoter region. 0.75 mM of L-histidine added to the preincubation mixture improved the protein-DNA interaction

To validate the computationally predicted DNA-binding motif of HisR, we heterologously expressed and purified the HisR protein from *Ruminococcus gnavus*. This anaerobic clostridium is a dominant community member in the human gut. Our genomic reconstruction suggests that *R. gnavus* contains three candidate HisR-binding sites located upstream of the *hisZGDB(FIE)* operon, hisXYZ* transporter and *hisR* regulator gene. A synthetic fluorescence-labeled DNA fragment containing the predicted HisR-binding site upstream of the *hisX* (Rumgna_0302) gene in *R. gnavus* was assessed for its specific interaction with the purified HisR protein using the fluorescence polarization assay. This synthetic 30-bp DNA fragment contained a 20-bp HisR site sequence flanked by poly-guanine stretches on each side. In the presence of an excess of histidine (0.75 mM), HisR specifically binds to the synthetic DNA fragment containing a HisR-binding site from *R. gnavus*. Exclusion of histidine from the incubation mixture led to the disruption of HisR-DNA binding (Fig. 6). Overall, this analysis of the *R. gnavus* HisR protein provided a sufficient experimental confirmation of the target HisR-binding sites and HisR-regulated genes tentatively identified by comparative genomics, while many aspects of the proposed mechanism are yet to be investigated. Experimental validation of candidate DNA binding motifs for HisR orthologs in other lineages of Firmicutes was beyond the scope of this mostly bioinformatics study.

## Conclusions

We present a bioinformatic analysis of transcriptional regulatory mechanisms for histidine biosynthetic and transport genes across the reference set of 626 representative genomes from the Firmicutes phylum. Identification of a novel DNA-binding regulator for histidine metabolism allowed us to fill a substantial gap in the knowledge of transcriptional regulation of amino acid metabolism in Bacteria. The HisR regulators belong to the TrpR family of transcription factors that control aromatic amino acid metabolism in *E. coli* and other g-proteobacteria [22]. HisR orthologs were found in all studied taxonomic groups of Firmicutes except for two orders: Erysipelotrichales and Lactobacillales. Additional HisR orthologs were found in several individual taxa of Proteobacteria (g- and a-proteobacteria), Actinobacteria, Synergistetes, Dictyoglomi, Elusimicrobia, and in one *Acholeplasma* species from the Tenericutes phylum. By using the comparative genomics approach that combines the identification of candidate regulator-binding sites with a cross-genomic comparison of regulons, we have reconstructed the HisR regulons in all above lineages that include the *his* biosynthetic operons/genes and/or one of two types of histidine transporters (Table 1 and Fig. 2). In nearly half of the analyzed genomes the *hisR* gene is preceded by a candidate HisR site suggesting autoregulation. The cognate DNA motifs of HisR orthologs have a common consensus in Firmicutes, Actinobacteria, and Synergistetes, whereas Proteobacteria possess a different HisR binding site consensus more resembling the TrpR binding motif (Fig. 3). We further selected a representative HisR regulator from *Ruminococcus gnavus* that belong to the Eubacteriales order of Firmicutes to experimentally assess its specific DNA-binding properties and test its potential effectors. Histidine was the most effective in stimulating the in vitro binding of HisR to its DNA operator. A comparison of the tertiary structures of TrpR, with a well-characterized mechanism of tryptophan-dependent DNA binding, with a recently available HisR structure revealed structural and sequence similarity between their C-terminal DNA-binding domains and key differences in their N-terminal domains that are involved in the effector binding (Fig. 4).

Additional analysis of regulatory regions of the histidine metabolism genes in Firmicutes lineages that lacked a HisR regulator allowed us to identify RNA regulatory elements of two different classes (Table 3). Histidine-specific T-box regulatory motif is the most common regulatory mechanism for the control of histidine metabolism genes in the Lactobacillales order, while its appearance in other lineages is quite rare. Novel RNA attenuators involving His-leader peptides, terminator, and antiterminator hairpins were identified in several taxonomic groups that lack both HisR and histidine T-box regulation. These observations allowed us to propose a plausible evolutionary scenario for the regulation of histidine biosynthesis genes in Firmicutes, which include the loss of an ancestral HisR-dependent regulation in the Lactobacilalles and some other lineages and its substitution with either histidine-specific T-boxes or RNA attenuators in certain taxonomic groups or individual species of Firmicutes.

This bioinformatic study presents comprehensive and detailed comparative analysis of regulation of histidine metabolism in bacteria supplemented by genome context and similarity searches. This study is a continuation of our previous analyses of amino acid-specific regulons [22, 29–31], that demonstrate the power of comparative genomics for the inference of both DNA- and RNA-level regulons and provide a unique possibility to study the evolution of regulatory systems in bacteria. We also successfully performed experimental validation of HisR regulon in a single species. We believe that further experimental testing of novel HisR regulators and RNA attenuators will shed light on their regulatory mechanisms.

## Methods

### Selection of analyzed genomes

All genomes used in this study were taken from the SEED database of bacterial genomes [32]. Among 1588 genomes from the Firmicutes phylum available in the SEED database in June 2019, we selected 626 genomes using the following criteria: if there are multiple genomes for the same taxonomic species, we chose a single representative strain with preference to include the type strains (e.g. from the ATCC or DSM strain collections). The obtained reference set of Firmicutes genomes was classified into eleven taxonomic orders including: Acidaminococcales (4 genomes), Bacillales (137 genomes), Eubacteriales (195 genomes), Erysipelotrichales (20 genomes), Halanaerobiales (7 genomes), Lactobacillales (174 genomes), Natranaerobiales (1 genome), Selenomonadales (16 genomes), Thermoanaerobacterales (43 genomes), Tissierellales (18 genomes), and Veillonellales (11 genomes). In addition to Firmicutes, we have collected bacterial genomes from other bacterial phyla containing HisR orthologs that include Proteobacteria (62 genomes), Actinobacteria (22 genomes), Synergistetes (9 genomes), Dictyoglomi (2 genomes), as well as Elusimicrobia and Tenericutes (a single genome in each group).

### Reconstruction of histidine metabolism subsystem and HisR regulons

The comparative genomic analysis of histidine biosynthesis, transport and regulatory genes was performed using the subsystems approach implemented in the SEED database and analysis tool, which combines protein similarity search, positional gene clustering, and phylogenetic gene profiling [32]. The Histidine metabolism subsystem includes eleven biosynthetic enzymes, the HisR repressor and four transporter genes encoding components of two histidine uptake systems (Table 1). Gene orthology was defined by the bidirectional best-hit criterion using blastp within the SEED database [32]. Orthologs of *hisR* genes were confirmed by the construction of the HisR protein phylogenetic tree. Multiple alignment of HisR proteins was built using MUSCLE [33] from the MEGA X software package [34] with the following settings: gap open, -2.90; gap extend, 0.00; Hydrophobicity Multiplier, 1.20; cluster method, UPGMA. The phylogenetic tree of HisR proteins was constructed using a maximum-likelihood algorithm implemented in MEGA X [34], using 100 bootstrap replicates, Jones-Taylor-Thornton model, and uniform rates. The TrpR regulator from *Escherichia coli* (ATCC 8739) was included to the alignment and tree as an outgroup. The tree was exported in*.nwk* format and downloaded in the iTOL visualization system (https://itol.embl.de) [35]. For visualization of metadata (types of regulation) and taxonomy on the constructed trees we used well-described iTOL script language.

For the identification of DNA binding sites and reconstruction of HisR regulons, we applied the integrative comparative genomics approach as previously described [24]. Briefly, to find conserved HisR-binding sites in each taxonomic group of the *hisR*-containing genomes we used the training sets of non-coding regulatory regions of the histidine subsystem genes. For training sets, we collected up to 300 nucleotides upstream of the translation start site and excluded the coding regions of any upstream gene if the intergenic region was less than 300 nt. The obtained training sets for each HisR-containing bacterial lineage were used for a search of lineage-specific conserved DNA motifs possessing a palindromic structure using the SignalX software [17] (http://bioinf.fbb.msu.ru/SignalX/), resulting in the construction of respective PWMs. The obtained lineage-specific PWMs were further used to scan the studied genomes and identify additional HisR-binding sites using the z-scan script and the GenomeExplorer software [36]. Scores of candidate sites were calculated as the sum of positional nucleotide weights. Specifically, we searched the upstream gene regions of histidine biosynthesis genes, putative histidine transporters and *hisR* genes, and reported all candidate binding sites with scores above a PWM-specific threshold (typically between 3.5 and 4.0). The search parameters were selected allowing identification of potential sites between 350 nt upstream and 50 nt downstream of a gene start codon. Cross-species comparisons of the predicted sets of potentially regulated genes allowed us to tentatively define regulon composition for each analyzed lineage. Candidate HisR binding sites with scores below threshold were validated by phylogenetic footprinting using multiple sequence alignment of upstream gene regions for each group of orthologs, as previously described in [17]. Multiple alignments of DNA upstream regions (Additional file 5) were obtained by MUSCLE from MEGA X [34], using unweighted pair group method with arithmetic mean.

### Identification of RNA regulatory motifs

Known RNA regulatory elements were identified using probabilistic covariance models that describe a combination of RNA secondary structure and sequence consensus. The covariance models of the analyzed RNA motifs, namely T-box (RF00230) and His leader (RF00514), were taken from Rfam database [26]. The 1000 bp upstream regions of histidine synthesis and transport genes were extracted from the SEED-annotated genomes. A scanning of gene upstream regions using these covariance models was performed by the *cmsearch* program from the Infernal package 1.1.3 [37]. Outputs of *cmsearch* with alignments of 157 candidate histidine-specific T-boxes in Firmicutes are provided in Additional file 8. Additional T-boxes were identified using Riboswitch Scanner [38]. Novel RNA regulatory structures serving as potential histidine attenuators were identified for those *his* genes that are not regulated by any above RNA motif or by a HisR repressor. To identify potential novel histidine attenuators in the genomes that lack known RNA motifs and HisR regulation, we generated multiple alignments of upstream regions of the *his* genes from target groups of closely related genomes (Additional file 6). RNA secondary structures of potential attenuator, terminator and antiterminator RNA conformations were predicted using Mfold [39]. To identify histidine leader peptides, the upstream regions presumed to contain an attenuator were searched for short open reading frames using UGENE [40].

### Phylogenetic tree of Firmicutes species

To construct a phylogenetic tree of the analyzed Firmicutes species, we used two groups of the following ribosomal proteins: L5, L6, L9, L10, L15, L20 and S2, S4, S5, S6, S8, which represent small (S) and large (L) ribosomal subunits, respectively. These eleven ribosomal proteins were identified in the analyzed 626 genomes using subsystems in the SEED database [32]. Multiple protein sequence alignments were obtained for each group of orthologous ribosomal proteins using MUSCLE in MEGA X [34] with the same settings as described above. The obtained alignments were further concatenated using SEDA 1.3 multitool with default settings [41]. The phylogenetic species tree was constructed using the concatenated alignment of eleven ribosomal proteins via the maximum-likelihood algorithm implemented in MEGA X [34], using 100 bootstrap replicates (see above) and visualized by iTOL v4 [35].

### Experimental validation of HisR regulon in vitro

The *hisR* gene from *Ruminococcus gnavus* ATCC 29,149 (locus tag Rumgna_03779 or EDN76156.1 in GenBank, length 299 bp) was synthesized by GenScript Inc. with optimized codons for expression in *Escherichia coli* by IDT tool (https://www.idtdna.com/CodonOpt). The cloning of the synthesized *hisR* gene fragment into the pODC vector was performed using the SalI and NcoI restriction enzymes. The PCR fragment was purified and subjected to the second round of PCR using primers HisR-NcoIF-GATTATCCATGGCTAAGAAAATCCGTACGG, and HisR-SalIR-CTAATTGTCGACTTATTTTTCCATGCGCTCAAAC, to append a six histidine codons and tobacco Etch Virus protease cleavage sequence. The fragment was digested with NcoI/SalI and ligated to the same sites of pODC. The resulting plasmid was introduced into *E*. *coli* DH5α to inoculate onto LB agar plates containing ampicillin. The colonies were isolated and checked by sequencing using verification primers. The HisR-pODC plasmid was introduced into *E. coli* BL21/DE3 strain used for overproduction of HisR protein.

The recombinant HisR-His_6_ protein containing an N-terminal 6 × His tag was overproduced by growing the BL21/DE3 strain carrying the plasmid in LB at 37 °C until mid-exponential phase (OD600 ~ 0.6), and 0.8 mM isopropyl-b-D-thiogalactopyranoside was added. The cultures were incubated at 24 °C overnight with continuous shaking, and cells were collected by centrifugation. The recombinant protein was purified by Ni-chelation chromatography from the soluble fraction as previously described [42–44]. The insoluble fraction was solubilized in 7 M urea and purified on a Ni–NTA minicolumn with At-buffer (50 mM Tris–HCl buffer, pH 8, 0.5 mM NaCl, 5 mM imidazole, and 0.3% Brij) with 7 M urea. The protein size, expression level, and purity were monitored by SDS-PAGE. Expected molecular weight of the recombinant HisR-His_6_ protein is 11.79 kilidaltons, with total length 100 amino acids. Protein concentrations were measured using the Bradford assay kit (Bio-Rad).

Interaction of the purified recombinant HisR repressor protein with its cognate DNA-binding sites in *R. gnavus* was assessed using fluorescence polarization assay (FPA), as previously described [45]. We selected to test a high-scored 20-bp binding site upstream of the *hisX* gene (Rumgna_00302), CAGTTTAGTATAGTAAAGT, that was linked to the GGGGG sequences at both ends to improve annealing of single stranded DNA fragments. The single stranded labeled and unlabeled DNA oligos were synthesized by Integrated DNA Technologies. The double-stranded DNA fragments were obtained by annealing synthesized oligonucleotides at a 1:10 ratio of 5’-labeled with 6-carboxyfluorescein to unlabeled complementary oligonucleotides. The obtained 30-bp DNA fragments (1 mM) were incubated with the increasing concentrations of HisR for 20 min. The HisR binding assay mixture (0.1 ml) contained Tris buffer, pH 7.5, 0.1 M NaCl, 0.5 mM EDTA, 10 mM MgSO4, 2 mM DTT, 5 mg/ml herring sperm DNA. Thereffect of histidine (0.75 mM) was tested by its addition to the incubation mixture.

## Supplementary Information

**Additional file 1.** Maximum-likelihood phylogenetic tree for the HisR proteins in studied genomes.

**Additional file 2.** Distribution of histidine biosynthesis genes, transporters and regulons in Firmicutes genomes.

**Additional file 3.** (A) Predicted HisR-binding sites and reconstructed regulons in other lineages; (B) Distribution of histidine biosynthesis genes and transporters in other lineages.

**Additional file 4.** (A) Predicted HisR-binding sites (A) and Histidine-specific T-boxes (B) and the reconstructed regulons in Firmicutes genomes.

**Additional file 5.** Phylogenetic footprinting of upstream regions of candidate HisR-regulated genes.

**Additional file 6**. Phylogenetic footprinting of upstream regions of genes controlled by candidate Histidine-dependent translational attenuators.

**Additional file 7.** Distribution of various regulatory mechanisms for histidine metabolism genes among analyzed Firmicutes species.

**Additional file 8.** Outputs of cmsearch (Infernal) with alignments of candidate histidine-specific T-boxes in Firmicutes.

## Data Availability

All data generated or analyzed during this study are included in this published article (and its additional information files). Protein Data Bank (PDB) access numbers used: 3G1C, 1ZT9.

## Abbreviations

RBS: Ribosomal-binding site
PWM: Positional Weight Matrix
HTH: Helix-turn-helix
Rmsd: Root mean square deviation
FPA: Fluorescence polarization assay
