# Supplementary material for: Evolution of transcriptional regulation of histidine metabolism in Gram-positive bacteria
Source: BMC Genomics. 2022 Aug 25;23(Suppl 6):558. doi: 10.1186/s12864-022-08796-y (PMC9413887; doi:10.1186/s12864-022-08796-y)

Additional file 7. Distribution of various regulatory mechanisms for histidine metabolism genes among analyzed Firmicutes species.
The maximum likelihood phylogenetic tree of species was constructed by MEGA X based on concatenated sequences of ribosomal proteins.
The size of circles in internal nodes represents bootstrap values in percentages (out of 100).
Colored bars show the occurrence of specific regulatory interactions in each species according to legend and Additional file 2.

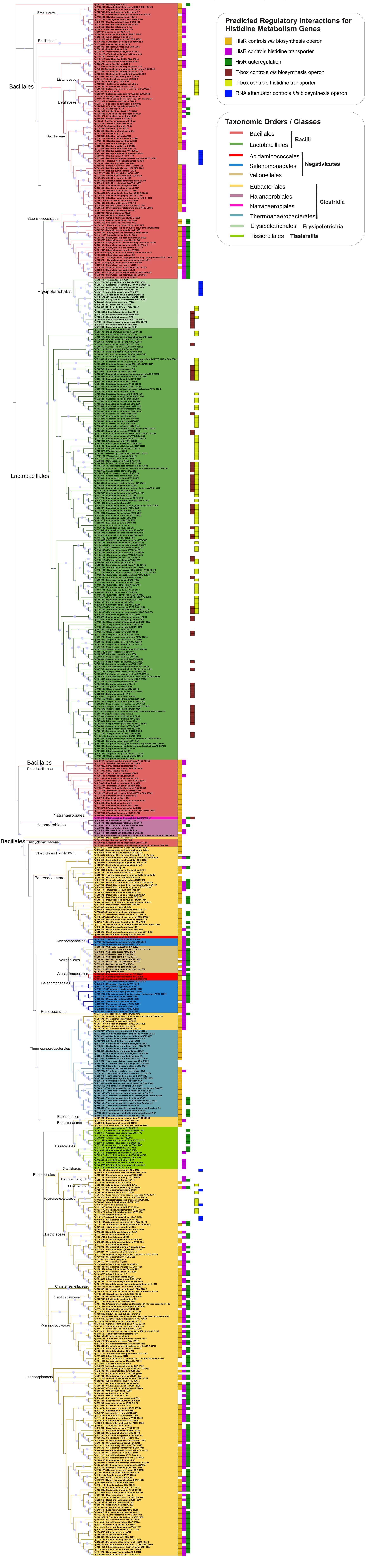

Supplement: Supplementary file 7 — Additional file 7. Distribution of various regulatory mechanisms for histidine metabolism genes among analyzed Firmicutes species. [file 12864_2022_8796_MOESM7_ESM.pdf]
