# Supplementary material for: Evolution of transcriptional regulation of histidine metabolism in Gram-positive bacteria
Source: BMC Genomics. 2022 Aug 25;23(Suppl 6):558. doi: 10.1186/s12864-022-08796-y (PMC9413887; doi:10.1186/s12864-022-08796-y)
Supplement: Supplementary file 5 — Additional file 5. Phylogenetic footprinting of upstream regions of candidate HisR-regulated genes. [file 12864_2022_8796_MOESM5_ESM.pdf]

Additional File 5. Phylogenetic footprinting of upstream regions of candidate HisR-regulated genes (see legend below).

fig|1196029.3.peg.4456 Bacillus endophyticus 2102
fig|1006007.3.peg.213 Bacillus megaterium WSH-002
fig|412384.9.peg.5136 Bacillus aryabhattai strain T61

```
fig|1196029.3.peg.4456 acagaaaatttgattatttcatatattatttgagta-tgaactgttgacgaggcgaaatgaaagatgtaaactactaataactttaattcattatttctc
fig|1006007.3.peg.213  gaactaagctgtggttttttttgtaa-atttgaacagctggcggttgacggctgactttatcgggtgtaaactatcaataactttaatttattatttcgc
fig|412384.9.peg.5136  gaactaagctgtggttttttttgtaa-atttgaacagctggcggttgacggctgactttatcgggtgtaaactatcaataactttaatttgttatttcgc
                         *  **  *    * ***  * **  ******  *     * *******        *    * *********  *************  ******* *

                          YRcwTTAgYRWRnTAAAGtR
fig|1196029.3.peg.4456 taattcgttagtgtgataaagtt-------------------tttttatt---ttaaatcaa------aggatg-gttacgATGTCGAAGTTATTTATGT 4.09
fig|1006007.3.peg.213  taatacattagtgtgataaagtaaaaacaacctatcaatggttccttaatagataaaatcaagcttaaaggacgtgtcggaATGTCAAAGTTATTTATGT 5.44
fig|412384.9.peg.5136  taatacattagcgtgataaagtaaaatcaacctatcaatggttccttaatagataaaatcaagcttaaaggacgtgtcagaATGTCAAAGTTATTTATGT 5.44
                       **** * **** **********                    *  *** *   * *******      **** * **    ***** *************
```

---

fig|1230341.3.peg.778 Salimicrobium sp. MJ3
fig|866895.3.peg.864 Halobacillus halophilus DSM 2266
fig|2045014.3.peg.1092 Halobacillus sp. Marseille-P3879

```
                                                   YRcwTTAgYRWRnTAAAGtR
fig|1230341.3.peg.778  tgtgaaagggtggatttttttatatttattctttatcatggtaaatcgttaat---------gagAT-------gaaaggggtggagatgATGGCATCGGCTGT 4.15
fig|866895.3.peg.864   aataagagggttgatttttttaatattgtactttattatggtaaatcgctaat---aaataaaagatagacgtagagaggagaatggattATGGCCTGGGCAGT 4.72
fig|2045014.3.peg.1092 aaaatgagggttgatttttttgaaataatcctttattatggtaaatcgctaatgtgataaaaagagtttaacttgagaggagattatagtATGGCACTAGCTGT
                             ***** *********    *  * ****** *********** ****                     ********     *  *****    ** **
```

---

fig|1036673.3.peg.173 Paenibacillus mucilaginosus KNP414
fig|717606.6.peg.3631 Paenibacillus curdlanolyticus YK9

```
                                  YWYWTTAnYwwRnTAAAGnr
fig|1036673.3.peg.173 ctaacgattacattcttcatcttggtagtatggtaacatattagcggactaaagcgttctgaaatattcatga  3.8
fig|717606.6.peg.3631 gtaataaggagtcgctttaacgcgatgaagcatcaacctaacagatga-tacgaattgatgataaatgaaaga  4.33
                       ***  *  *    *** * *  * *        *** **  **  ** **     *  *** * **  * **

fig|1036673.3.peg.173 ctaaacgaagatagat--agggtgaactccaTTGTCCAAACCGAAGGTGTTTGAGAAGCCTACAGGCGTGAAGGACT
fig|717606.6.peg.3631 acaaacacgattagaagaagggtgagtcccGT-GTCGAAACCAAAAGTATTTGAAAAACCGATCGGCGTTAAAGATT
                        ****     ****   *******   ** * *** ***** ** ** ***** ** ** *  ***** ** ** *
```

---

fig|742737.3.peg.2516 — **Clostridium hathewayi WAL-18680**
fig|610130.3.peg.2135 — **Clostridium saccharolyticum WM1**
fig|1304881.3.peg.4929 — **Clostridium indolis DSM 755**
fig|6666666.205263.peg.197 — **Hungatella hathewayi LFYP18**

```
                                                                                nRyTTTAnYRYRnTAAArYr
fig|742737.3.peg.2516      gattgacagatgtttacttttcacctataataataaccacgttatcgaattagcactgcgccatgcgaaagtgatgtgg----------------     3.75
fig|610130.3.peg.2135      gattgacaaggaacactttttcgcctataattgactacacgttatcgaattatcactttactacaagaaagcgaaataaattgacaagttgtgaa     4.42
fig|1304881.3.peg.4929     gattgacaagaaccactttttcccctataattgactacacgttatcgaattatcactttactataagaaagtggtaaaaatgatgagtcgtgaa
fig|6666666.205263.peg.197 gattgacaagtcgggcagtgtcccttataataaactacacgttatcatggtagcactttaccatatgaaagcagta-------------------
                           ********          * ** * ******      *********    ** ****   * *   *****

fig|742737.3.peg.2516      ----------------------------------cggtgagaaaaggtcaggcccgccagacTTGTTTGAGCGGGGCGGGTATCTGGCAGTGCAAA
fig|610130.3.peg.2135      aataattttctccactggttttatgacg-cttgtcatgaagatgtgaagcagaagacggaggttgttATGGCAAATAAACTGTTACATACACCGGA
fig|1304881.3.peg.4929     aataattttcttcactgcttttttgacggctcactgtggatatgcgaagcagaagacggaggttgttATGGCAAATAAACTGTTACATACACCGGA
fig|6666666.205263.peg.197 --------------------------------------------tgcagcaacgttcggaggaagctATGGCAAACAAATTATTACATACACCGGA
                                                                        *          *             **        * *     *   *  *
```

---

fig|626937.8.peg.496 — **Christensenella minuta strain DSM 22607**
fig|572545.4.peg.453 — **Ruminiclostridium thermocellum DSM 2360 strain LQRI**
fig|203119.11.peg.3083 — **Clostridium thermocellum ATCC 27405**

```
                                                                                   nRyTTTAnYRYRnTAAArYr
fig|626937.8.peg.496   gcttaattaatttgttgcttgacagaaaaaaatgtttatattattatagtagcgtgatattgatttactacgctaaattattaaagggatgaa------c   4.17
fig|572545.4.peg.453   atttttctcattgacaaattaatccacatgaattataataaccttagttcatcaatttataactttaacatgataaagtgataaagtaataaagtgaggt
fig|203119.11.peg.3083 atttttctcattgacaaattaatccacatgaattataataaccttagttcatcaatttataactttaacatgataaagtgataaagtaataaagtgaggt   5.16
                         **   * ***      ** *   * *  ***  * ***   ***    * *    ***   ****  * * **** *  *****  ** **

fig|626937.8.peg.496   gattatAT-GGAACAGTTAAAAACGCAGGTCCCCGCGGGCGCCTGGGACT
fig|572545.4.peg.453   gcttaaaTTGGCAGAGTGGAAGATATATACTCCTGAAGGTGTTCAAGATA
fig|203119.11.peg.3083 gcttaaaTTGGCAGAGTGGAAGATATATACTCCTGAAGGTGTTCAAGATA
                       * ***  * ** * ***  ** *   *    ** *  ** *     **
```

---

fig|573061.5.peg.4050 — **Clostridium cellulovorans 743B**
fig|941968.4.peg.1627 — **Clostridium botulinum H04402 065**
fig|471871.7.peg.1986 — **Clostridium sporogenes ATCC 15579**
fig|431943.8.peg.1355 — **Clostridium kluyveri DSM 555**

```
                                                             nRyTTTAnYRYRnTAAArYr
fig|573061.5.peg.4050 acattgacaca-agaataaaat-aataatataattttagtaatttaattagctaaagtgataaa---gtaaaacagggg-gcaattATGAAAAAATTAAA   4.06
fig|431943.8.peg.1355 agattaactgatagaatagcattaataatttaattt-agcactacattgaattaaagcgataaa---acaagg-aagtg-atttctATGGCTAATTGGAG   4.24
fig|941968.4.peg.1627 acagtagtaaaaagaaatgataatatatttttaattatcactttatccaaataaactaataaagtagtaaagcgagggaggcagaATGGAAAATTGGAA   5.38
fig|471871.7.peg.1986 acagtagtaaaaagaaatgataatatatttttaattatcactttactcagataaactaataaactaataaagtgaggg-gatagaATGGAAAATTGGAA   5.38
                      * * *     * ****        *** * * * ** *  * *  *   *  ****   *****     **     * *       ***   ** *  *
```

fig|693746.9.peg.2496 **Oscillibacter valericigenes Sjm18-20**
fig|1007096.3.peg.1934 **Oscillibacter ruminantium GH1**
fig|290055.3.peg.2835 **[Eubacterium] fissicatena strain KCTC 15010**

```
                                                                  nRyTTTAnYRYRnTAAArYr
fig|693746.9.peg.2496  tttttcattaaaaatttt-t-gatatccgttatt-tacagctataatttatcatggtacgctggtagcatattaaatcagagggacatgtccctcaatttt   3.89
fig|290055.3.peg.2835  ttcagaattgacaacagagcagaaatgtgttattatgtctaagcattttagcgtggtaaagtaat----caattagtaaaagaataaagcgccacggcaa-   5.37
fig|1007096.3.peg.1934 tcttccagaaaaaaattaac-gatatgcaatatt-tacaggattaatttatcatggtaaactgatagtgcattaaatcagagggcaatg-ccctcagtttt   4.80
                       *     *   * **       ** **    **** *        * **** * *****   *  *     * * * * * **    * *  ** *

fig|693746.9.peg.2496  ggaggatttatg-ATGAATACGAAAAAAATTTTGGCTCTGCTGAGTGCGGCGGCTGTC
fig|290055.3.peg.2835  ggaggacgtacctATGAAAAGAAAGATTGCATTAGCAATGGCAGCTGCACTTGTGCTG
fig|1007096.3.peg.1934 ggaggatttaag-ATGAATAGAAGAAAGATTTTGGCTCTGCTCAGCGCGGCGGCTGTC
                       ******  **   ***** *  *  *     ** **  **      **    *   *
```

fig|931626.3.peg.2360 **Acetobacterium woodii DSM 1030**
fig|53442.3.peg.1313 **Eubacterium callanderi strain NLAE-zl-G225**
fig|903814.3.peg.339 **Eubacterium limosum KIST612**

```
                                                                                  nRyTTTAnYRYRnTAAArYr
fig|53442.3.peg.1313  gattaaattttcctaagaatattgaccttttataaaataaatgttattatatcaccacattactttattgcgctactgcaataaatcatcggaggaa--a   4.21
fig|931626.3.peg.2360 gtttaaaaacaattaaaaggtttgacaaacta-aaaataaatgatacaatagttttataccacttcactagactacagtaataaagcagttgttgaacaa   4.16
fig|903814.3.peg.339  gattaaattttcctaagaatattgaccttttataaaataaatgttattatatcaccacatcactttattgcgctactgcaataaatcatcggaggaa--a   4.30
                      * *****      *** *   *****    ** ********** **  ***     * *  **** * *   **** * ****** **   *  ***  *

fig|53442.3.peg.1313  gca-------ATGCTCGAAAATTACACGATTGATGGCTTTGAAAATATTC
fig|931626.3.peg.2360 ggaggttcttATGCTTTTTAATTATACCATTGACGGCTTTGAAAATATAC
fig|903814.3.peg.339  gca-------ATGCTCGAAAATTACACGATTGATGGCTTTGAAAATATTC
                      * *       *****    ***** ** ***** ************** *
```

fig|1408436.3.peg.1775 **Eubacterium cellulosolvens LD2006**
fig|1123075.3.peg.1485 **Ruminococcus gauvreauii DSM 19829**

```
                                                                    nRyTTTAnYRYRnTAAArYr
fig|1408436.3.peg.1775 ggggccggaattgtcaggacttgttatagaaaaaggttgacatgaaaatttgttatggtaatatggtagcagactaaagtgcaaaagcgcgagagtgaat   4.24
fig|1123075.3.peg.1485 tgtccagggattgaca--ttttattcaggatggactataatcgaaaagattcacatggtaatatgctaccatggtagcactttaatacgataaagc----   4.83
                        *  * ** **** **    ** **   **   *   * *    ***  **   *********** ** **   **       **  **  * **

fig|1408436.3.peg.1775 agacgaagaggagtacaatcATGAAAAAACTCATACATACACCGGAAGGTGTCCGCGATAT
fig|1123075.3.peg.1485 agaaggagtatatgaaaATGGTACAAAGAATGTTCCACACGCCGGAGGGCGTACGTGATGT
                       *** * **   *  * *    *  *** * *  * ** ** ***** ** ** ** *** *
```

fig|1414720.3.peg.2641 Clostridium sp. JCC
fig|931276.5.peg.1495 Clostridium saccharoperbutylacetonicum N1-4(HMT)

```
                                                                              nRyTTTAnYRYRnTAAArYr
fig|1414720.3.peg.2641 aataattgacaaataaaaccatata----aggtattattgataataactt--taacactttaatctgctaaaggg-ggcgatttgaattaaaaatattat   4.24
fig|931276.5.peg.1495  aatttttagaattattgacatgcaatagaaaatactatattataaatttaattacagtttagcacgttaaagcgataaaatataaatggataatagtgg   4.82
                         *  ** * ** **      *  *    *        *   *  **  *     ** ****    * ***** *     *  * **      * * *

fig|1414720.3.peg.2641 accagaaggaATGCGAGATTTTACGG
fig|931276.5.peg.1495  gg--tggaaaATGAGTAAGAAGAATA
                                **** *  *    *
```

---

fig|1871029.3.peg.2807 Intestinibacillus massiliensis strain type strain: Marseille-P3216
fig|2364792.3.peg.822 Intestinibacillus sp. Marseille-P6563

```
                                                          nRyTTTAnYRYRnTAAArYr
fig|1871029.3.peg.2807 cccgcagccgcctttttttg---caaaatttttattgattttcttgaataaagtggt-acaatattaaaa-gactgttcatttaaactttagga-----   4.07
fig|2364792.3.peg.822  tacaaaaattctttggatcgactcgattttgctgttgattttttcgaactaaattgctacaataataacacggttcttgaaaagggatagtagaaagcgg   4.50
                         *  *    * **   * *        **  * ******** * ***  ** * *  ****** *** * *    ** *      *   *** *

fig|1871029.3.peg.2807 ---ggactatacttATGAAGATGAAGCGCATTGCAGGCCTGGTGCTGGCC
fig|2364792.3.peg.822  aaaggaatgtacttATGAAAGCAAAGAGACTCTTCAGCCTGCTTCTGGCG
                          *** * **********      * *  *     ***** * *****
```

---

fig|1121323.3.peg.2212 [Clostridium] lactatifermentans DSM 14214
fig|991789.4.peg.2525 [Clostridium] propionicum DSM 1682
fig|1123282.4.peg.2032 Sporobacter termitidis DSM 10068

```
                                                                                 nRyTTTAnYRYRnTAAArYr
fig|1121323.3.peg.2212 aaatatctgaataaaaacttagcaatttgtatatacataccaaactttaataatctacttgactaaagtgctaaactggcttaatatgaagaaaagca   3.70
fig|991789.4.peg.2525  aaaaaaatagaaagataaaaacgagtttcagcaaagatgataaactttagtgttctacttgactaaagtggtaaactgatataagattaaagtaaatt   3.76
fig|1123282.4.peg.2032 aaaaaaagacgcagaacaacttcgatgccaatgaat-tatcata--ttaccgaattacttgactaatatgctaaagtatcgtatactgatgtcaccaa   3.76
                       *** *       * *          *        *  *   * *  ***      ***********  ** **** *    **   * *    *

fig|1121323.3.peg.2212 tatg---ggaggtgccttATGACTGATTGGGATGGATTATTGAAAAAAGAAGAACAGACGGTGT
fig|991789.4.peg.2525  gacgtgcggaggtgcaacATGACATTGCCGAAAATGCTTTTGAAAAAAGAAGAGGAAGTTATAT
fig|1123282.4.peg.2032 cagaaagtgagaaagagcATGACCGTTTCAGAGGATGTCCTGAAAAACGAGGAGAAGGCGATGA
                        *      ***       *****        *     *  ******* ** **  *     *
```

fig|476272.5.peg.243 **Blautia hydrogenotrophica DSM 10507**
fig|411459.7.peg.1542 **Ruminococcus obeum ATCC 29174**
fig|1095771.3.peg.1573 **Ruminococcus sp. JC304**

```
                                                                                  nRyTTTAnYRYRnTAAArYr
fig|476272.5.peg.243   gggattgacttttcagcatttttcttataatctaactaatttgttttatgtgctaatatagtagcgagataaagtactgaatt--aacg---------   4.17
fig|1095771.3.peg.1573 g--attgacattttcagaataaaccagtataatttta----tctagtttatgcatcactgtagtaacatggtagcatgctaaagcaaaatgg--------
fig|411459.7.peg.1542  gggattgacattttctattttttctactataatttt-----cttgttttacatagtaacatggtagcacgttaaagtgcttatctgtaatga--------   4.87
                           ***** ****                 ******                *   ***           *   * *** *   * **    * ** *      **

fig|476272.5.peg.243   -------------------------ggagagaatggatA-TGGAAAGAATTTTTCACACACCAGAGGGAGTCAGGGATATCTGCCAGGAA
fig|1095771.3.peg.1573 -----------aagaca--------aggaggatcacgcaATGGAGCGTATTTTTCATACACCAGAGGGTGTAAGGGATATTTACAATGGT
fig|411459.7.peg.1542  -------------------------ggaggaaacgactA-TGCAGCGAATTTTTCATACACCTGAAGGTGTTCGCGATATTTATAATGGA
                                                  *    *         ** *  * ***** ** ** ** ** ** **   * ** ** *    * *
```

---

fig|1410649.3.peg.2004 **Blautia schinkii DSM 10518**
fig|657314.3.peg.2472 **Ruminococcus obeum A2-162**
fig|40520.1561.peg.1222 **Blautia obeum strain af_0058**

```
                                                                          nRyTTTAnYRYRnTAAArYr
fig|1410649.3.peg.2004  ggaatccaattagagaggaaaagtgggattgacttatgtaatgcactatggtagtatggtagcacaataaagcattggttgtaagtaagacacacgag 4.26
fig|657314.3.peg.2472   ggattgacaatttgggtttttcctattat-aatttctttgtttt-atatagtagcatggtagcacgttaaagcattc--tgcctacaa------tgag
fig|40520.1561.peg.1222 ggattgacattttctatttttttctactat-aattttcttgtttttacatagtagcatggtagcacgttaaagtgtt---tatctgtaa------tgag
                            *     *                  *  * *        *      ** **** **********  *****  **   *       **       ***

fig|1410649.3.peg.2004  gaggaaatagacATGCAGCGAATTTTTCATACACCGGAGGGTGTACGGGA
fig|657314.3.peg.2472   gaggaaccgattATGCAGCGAATTTTTCACACGCCGGAAGGTGTACGCGA
fig|40520.1561.peg.1222 gaggaaacgactATGCAGCGAATTTTTCATACACCTGAAGGTGTTCGCGA
                        *****       ******** ***** ** ** ** ** *****  * **
```

---

fig|657324.3.peg.1663 **Butyrivibrio fibrisolvens 16/4**
fig|1123011.4.peg.475 **Pseudobutyrivibrio ruminis DSM 9787**

```
                                                                                   nRyTTTAnYRYRnTAAArYr
fig|657324.3.peg.1663 gtttttttacgccctgaggccattgttgacttcggggctttattaaagtataataactttagttctttatcagactaaagtgt---tttttataagaggg   4.20
fig|1123011.4.peg.475 gaaatagagtgccccgaggccat-gttgacttcggggctttattaaagtataataactttagttctttatcacgctaaagtgtagttttgtacaagaggt   4.78
                      *   *     **** ******** ***********************************************   *********    *** ** ******

fig|657324.3.peg.1663 acaaaaATGAAAAAATCTTACTTTACACACTCCAGAGGGAGTAAGAGATAT
fig|1123011.4.peg.475 gcgaaA-TGAAAAAATTTAACTTTACACACTCCAGAAGGAGTTCGAGACAT
                       * **  ******** * ***************** *****  **** **
```

fig|717962.3.peg.1595 **Coprococcus catus GD/7**
fig|180332.6.peg.2784 **Robinsoniella peoriensis strain 6600698**

```
                                                                                        nRyTTTAnYRYRnTAAArYr
fig|717962.3.peg.1595    caggccaaatgacatcacttgacaattgtggaaaggtatattataattagtttcacgtattatcaaattagcagactaaagcgtttgcg----------- 4.27
fig|180332.6.peg.2784    ggattgactttttttcatacatctattataatgaaaaataattttat-atagtagtgaattatcacgttaatgtgataaagtgaaacgaaatttgatttt 4.94
                               *                   * *        * *   *    *   *     *******  ***      ***** *

fig|717962.3.peg.1595    gtaaaggagcctatt---ATGG---ATAGTTTATTACATACTCCGGAAGG
fig|180332.6.peg.2784    cagaaagaggagagagcaATGAATCAACGATTATTACATACACCGGAAGG
                            ** ***         ***    *     * ** ** ** ***** **
```

---

fig|717962.3.peg.947 **Coprococcus catus GD/7**
fig|290055.3.peg.2835 **[Eubacterium] fissicatena strain KCTC 15010**
fig|2219695.3.peg.1831 **Clostridiales bacterium Marseille-P5551**

```
                                                            nRyTTTAnYRYRnTAAArYr
fig|717962.3.peg.947   ttcttgacaaaaacatatctgtatgttatcatgatagcgcgttagtagagtaaagagagtgaaccgtgaaaa-c------------gtaagatg   3.85
fig|290055.3.peg.2835  aacagagcagaaatgtgttattatgtctaa------gcattttagcgtggtaaagtaatcaattagtaaaagaataaagcgccacggcaaggag   5.37
fig|2219695.3.peg.1831 atcagggcagaaatatgttattatgtccag------gaattttaacgcggtaaagtaatcaattgataaaagaataaagcgccgcggtgaggag
                              ** ***  * *   *****          *    ***     ******  *   *    * ***               *  **  *

fig|717962.3.peg.947   gaggcat-ttATGAAAAAGGGTTTAGCTTTATGCATGGCTGCAGTATTAG
fig|290055.3.peg.2835  gacgtac-ctATGAAAAGAAAGATTGCATTAGCAATGGCAGCTGCACTTG
fig|2219695.3.peg.1831 gacgtacattATGAAGAAGAGATTAGCAGCAGCATTAGCGGCGGCCCTTG
                       ** * *   ****** *      * **        * ** **     * *
```

---

fig|1432052.14.peg.3960 **Eisenbergiella tayi strain DSM 26961**
fig|658086.3.peg.913 **Lachnospiraceae bacterium 3_1_57FAA_CT1**
fig|290055.3.peg.2835 **[Eubacterium] fissicatena strain KCTC 15010**

```
                                                                           nRyTTTAnYRYRnTAAArYr
fig|1432052.14.peg.3960 tgcttttgtattgacatatgaaatctcttcatgataggataatgccagcggcagtttagcctgataaactgctaaattactaaagaagcagtgcgatgca   4.11
fig|658086.3.peg.913    tgcttttgtattgacatatgaaatctcttcgtgataggataattccagcggcagtttagtctgataaactgctaaattactaaagaagcagtgcgatgca
fig|290055.3.peg.2835   caggcgcatttctgcttcagaattgacaacagagcagaaatgtgttatta-tgtctaagcattttagcgtggtaaagtaatcaattagtaaaagaataaa   5.37
                                *           ** *  *        ** *   *   *        * **  *  **   ** **** ** * **   * *     **  *

fig|1432052.14.peg.3960 ttgtttccggcatgaggaggagaaaattATGAAAAAGGCAGTTGCAATTTTGCTGGCAGGCATGATGGTA
fig|658086.3.peg.913    ttgtttccggcatgaggaggagaaaattATGAAAAAGGCAGTTGCAATTTTGCTGGCAGGCATGATGGTA
fig|290055.3.peg.2835   gcgccac-ggca--aggaggacgtacctATGAAAAGAAAGATTGCATTAGCAATGGCAGCTGCACTTGTG
                              * ****   *****    *  ****** *      * *** *     ******      * **
```

fig|887325.3.peg.962 **Eubacterium saburreum DSM 3986**
fig|467210.3.peg.308 **Lachnoanaerobaculum saburreum strain DNF00896**
fig|575593.3.peg.2148 **Lachnospiraceae oral taxon 107 str. F0167**

```
                                                            nRyTTTAnYRYRnTAAArYr
fig|887325.3.peg.962  gagtcaaagagtgctacaagtctgaataaagcagaaatactttaatgtagtagagaaacaaagtgttgacaagaacactttagtatgctaccataagaaagt   4.27
fig|467210.3.peg.308  gaatcaaagggatctacaaatcagaacaaagcagaattactttagtgtagtagagaaataaagtgttgacagatacactttagtgtgctaccataagaaagt
fig|575593.3.peg.2148 ataatggctctttttattaaattaaatgaa-tataaaaactttatttagtagagaaataaagtgttgacaaaactactttagcatgctaccataagaaagt
                                    **   *    **  **  *  ** ****** * *********** ************     *******  *****************

fig|887325.3.peg.962  gttgaggcaaagcaa--------agaaagtaatgaggtgtattATGAAAAAAATTTATTTGTTTGGAGTATTGGCGGCACTTA
fig|467210.3.peg.308  gttaagacaaagcaacaaagcaaagaaagtaatgaggtgtattATGAAGAAAATTTATTTGTTTGGAGTATTGGCGGCACTTA
fig|575593.3.peg.2148 acaaaggaacattaaaaaagcaagcgaaaaaatgaggtgtattATGAAAAAAATGTATTTATTGGGAATTTTGGCAGCATTTG
                          **  * *  **           **  ****************** ***** ***** ** *** * ***** *** **
```

fig|1291051.3.peg.2211 **[Clostridium] glycyrrhizinilyticum JCM 13369**
fig|658655.3.peg.174 **Lachnospiraceae bacterium 1_4_56FAA**
fig|290055.3.peg.1890 **[Eubacterium] fissicatena strain KCTC 15010**
fig|411470.6.peg.2154 **Ruminococcus gnavus ATCC 29149**

```
                                                                                                                     nRyTTTAnYRYRnTAAArYr
fig|1291051.3.peg.2211 agaaagatctggatgagcgggaagaactcgggattgacatttagtcggaaaattcgtataatg-actaaagattttaacgtagtagcagagtaaagtgcg   4.01
fig|290055.3.peg.1890  agaaggatttggat-----------accagggattgacattttctgagaaactgcgtataatttacagggtgatttaacgaattagcatgataaagtgaa   4.70
fig|411470.6.peg.2154  aaaaagatttggat-----------gacagggattgacagaagaaatgaaagttcgtataatt-acagaatgatttaatgtattagcatagtaaagtgaa   4.79
fig|658655.3.peg.174   agaaagatctggatgagcgggaagaactcgggattgacatttagtcggaaaattcgtataatg-actaaagattttaacgtagtagcagagtaaagtgtg
                       * ** *** *****               **********        **** * ********  **        ***** * * *****   *******

fig|1291051.3.peg.2211 gaggtgttgag----ATGGAACAAAAATTACATACGCCGGAGGGTGTAAGAGATATTTACAGTACA
fig|290055.3.peg.1890  agg----caggaaatATGGAACAGAAATTACATACACCTGAAGGCGTCAGGGATATATACAATACA
fig|411470.6.peg.2154  agggtgttatgaattATGGAACAAAAGTTACATACTCCCGAAGGAGTTCGAGATATTTACAGTAAT
fig|658655.3.peg.174   gaggtgttgag----ATGGAACAAAAATTACATACGCCGGAGGGTGTAAGAGATATTTACAGTACA
                         *       *    ******** ** ******** ** ** ** **  * ***** **** **
```

fig|693746.9.peg.4538 **Oscillibacter valericigenes Sjm18-20**
fig|1007096.3.peg.459 **Oscillibacter ruminantium GH1**

```
                                                     nRyTTTAnYRYRnTAAArYr
fig|693746.9.peg.4538 aaaggggaaatttaccccgtagaaaaaaatgtactttactttgatatctggatgaagtattataaaatcattaaactgtccgacatgggcggacaacgga   3.66
fig|1007096.3.peg.459 aaaagaggaaattccgccatataaaaaaatgtactttactttgataacaagatgaagtatgataaatccattaaactgtccgatataggcggataacgga   4.07
                      *** * * ** ** * ** ** ************************ *  ********** *****  *************** ** ****** ******

fig|693746.9.peg.4538 gggacatATGGAGTTGGATCTGGGAATATTGAAACCGCAGGAGC
fig|1007096.3.peg.459 gggacatATGGAGCTGGATCTGGGGATATTGAAACCGCAGGAGC
                      ************* ********** *******************
```

```
fig|756499.4.peg.3744       Desulfitobacterium dehalogenans ATCC 51507
fig|146907.3.peg.1309       Desulfitobacterium sp. PCE1
fig|871963.5.peg.300        Desulfitobacterium dichloroeliminans LMG P-21439

                                                                               nRyTTTAnYRYRnTAAArYr
fig|756499.4.peg.3744 attaaaaaactattgccagggaaaaatgagattgctataataaacataacgcgctaacactttaattagttaaagtgatgaaatgagtaaagaggagttg   4.16
fig|146907.3.peg.1309 attaaaaaactattgccagggaaaaatgagattgctataataaacataacgcgctaatactttaattagttaaagcgatgaaatgagtaaagaggagttg
fig|871963.5.peg.300  ttttgaaaagtattgccaaggtaggtagaggttgctataataactgtaatgctctaacgctttaactaactaaagtatttttata--tattgaggagatg   4.37
                            * *  * *   * *       **  *     ******   *** ** ****  ****** **  *****   *   **   **  ****** **

fig|756499.4.peg.3744 agggaggTTGGCTCGGAATTTCTTAACCATCGCTCTTCCTAAAGGAAAAT
fig|146907.3.peg.1309 agggaggTTGGCTCGGAATTTCTTAACCATCGCTCTTCCTAAAGGAAAAT
fig|871963.5.peg.300  agagagaTTGGCTCGTAATTTCTTAACCATCGCTCTGCCAAAGGGTAAAT
                      ** *** ******** ******************** ** ** ** ****
```

```
fig|138119.41.peg.4375      Desulfitobacterium hafniense Y51
fig|756499.4.peg.3744       Desulfitobacterium dehalogenans ATCC 51507
fig|537010.4.peg.4891       Desulfitobacterium hafniense DP7
fig|871963.5.peg.300        Desulfitobacterium dichloroeliminans LMG P-21439

                                                                             nRyTTTAnYRYRnTAAArYr
fig|138119.41.peg.4375 aaaaaactattgccagggaaaaatgaggttgttataataaccataatacgctaatgctttaactaattaaagtgatgaaatacatgaagaggagttgaga   4.10
fig|756499.4.peg.3744  aaaaaactattgccagggaaaaatgagattgctataataaacataacgcgctaacactttaattagttaaagtgatgaaatgagtaaagaggagttgagg   4.16
fig|537010.4.peg.4891  aaaaaactattgccagggaaaaatgaggttgttataataaccataatacgctaatgctttaactaattaaagtgatgaaatacatgaagaggagttgaga
fig|871963.5.peg.300   tgaaaagtattgccaaggtaggtagaggttgctataataactgtaatgctctaacgctttaactaactaaagtattttttatatatt--gaggagatgaga   4.37
                         **** ******** ** *    *** *** ********   ***  * ****  ****** **  ******  *   **   *   ****** ****

fig|138119.41.peg.4375 gagaTTGGCTCGGAATTTCTTAACCATCGCTCTTCCTAAAGGAAAACTAT
fig|756499.4.peg.3744  gaggTTGGCTCGGAATTTCTTAACCATCGCTCTTCCTAAAGGAAAATTAT
fig|537010.4.peg.4891  gagaTTGGCTCGGAATTTCTTAACCATCGCTCTTCCTAAAGGAAAACTAT
fig|871963.5.peg.300   gagaTTGGCTCGTAATTTCTTAACCATCGCTCTGCCAAAGGGTAAATTAT
                       *** ******** ******************** ** ** ** *** ***
```

fig|485916.5.peg.755 **Desulfotomaculum acetoxidans DSM 771**
fig|1121428.3.peg.2623 **Desulfotomaculum hydrothermale Lam5 = DSM 18033**
fig|696281.4.peg.1365 **Desulfotomaculum ruminis DSM 2154**
fig|868595.4.peg.1894 **Desulfotomaculum carboxydivorans CO-1-SRB**
fig|696369.3.peg.2190 **Desulfotomaculum nigrificans DSM 574**
fig|349161.6.peg.2584 **Desulfotomaculum reducens MI-1**
fig|635013.3.peg.889 **Thermincola sp. JR**

```
                                                                           nRyTTTAnYRYRnTAAArYr
fig|485916.5.peg.755   aataagca-ttgacagccccggaagagttgatttattattatagcgtattaatgctttaatatagtaaagaggtgaagtga  4.15
fig|1121428.3.peg.2623 aattagtacttgacaccaccttgcta-tcaatttaacataatattatattagtaatttaacaagttaaagaggtggt-tta  4.09
fig|868595.4.peg.1894  aaaaagttcttgacaccatcataatg-ctattttaccatatcattatgttagtaatttaataaactaaagggggccgggtt  4.26
fig|696369.3.peg.2190  aaaaagttcttgacaccatcataatg-ctattttaccatatcattatgttagtaatttaataaactaaagggggccgggtt  4.26
fig|696281.4.peg.1365  aaaaaactattgacagcagcaagatg-ctattttaacatattattatattactaatttaattaactaaaggagggaccgcc  4.10
fig|349161.6.peg.2584  aaattttacttgacacctttactacatgcat-ttaacatatcattatattagtaatttaataagttaaagaggtgataccc  4.05
fig|635013.3.peg.889   taccttggattgacatttat-caaaatccatgctataatttcatcatgttagcgctttaacgcgctaaagcgaaaggggtc  4.82
                        *       ******                  **  **   *   * ***    *****     *****

fig|485916.5.peg.755   tTTGGATTCTTCCAGG-GGGACTTTGACCATAGCCATACCTAAAGGGACTTTA
fig|1121428.3.peg.2623 tTTGGTGGCGGCCAGG-GAGCTGTTAACCATTGCTCTGCCCAAAGGGACTCTC
fig|868595.4.peg.1894  tTTGGTTGCTGATAGA-GATATTTTAACCGTGGCCTTGCCAAAGGGCACACTT
fig|696369.3.peg.2190  tTTGGTTGCTGATAGA-GATATTTTAACCGTGGCCTTGCCAAAGGGCACACTT
fig|696281.4.peg.1365  TT-GGTTGCGGCCAGA-GATATTTTAACAGTGGCTTTGCCCAAAGGGACCCTT
fig|349161.6.peg.2584  GT-GGTGGCAGCCAGA-GAACAATTAACTATTGCTTTGCCCAAGGGAACCCTA
fig|635013.3.peg.889   ct-ggtgttttATGGATGATTATTTATCTATAGCTATTCCCAAAGGGACACTA
                        * ***        *  *     **  *  * **  * ** ** ** **  *
```

---

fig|457570.14.peg.2058 **Natranaerobius thermophilus JW/NM-WN-LF**
fig|555088.4.peg.1367 **Dethiobacter alkaliphilus AHT 1**
fig|574087.3.peg.1493 **Acetohalobium arabaticum DSM 5501**

```
                                                                                          nRyTTTAnYRYRnTAAArYr
fig|457570.14.peg.2058 tacagagacccattttattgacggaataaaataggcgtgataagatattaccaat--gattaattaacactttaacgagttaacgaaataaaggaggttc   3.88
fig|574087.3.peg.1493  tgttaggatt--ttctatatttatattgacatttaaaaattagtatgttatattt-taataagttaatactttactgtattaaaataataaag--aggtg   5.08
fig|555088.4.peg.1367  ggtaaaggtt--gt-taactttgtactaaaattattgacacggcgccttgtgcatgttaaaatttaatacgttatcactttaacgtattaaagcggggta   5.02
                             *      * **       * * * **               **     *   *  * **** ** ***     ****   * *****   * *

fig|457570.14.peg.2058 cttaaTTGGTCCCAAGAGAGATTCAGCTGTTACAAAAACCATCAGGAGTCAACGAT---
fig|574087.3.peg.1493  gtagaATGGA---AAGTGAATTGAATTTTACTAAGATGCAGACCCCCGATGGTACTAAG
fig|555088.4.peg.1367  tctacATGAA---TGATAAAATGAAACGACTGCTGACGCCGGAAGGTGTCCGCGATTTG
                             **          *  *  *          *  *        *       *
```

fig|589861.3.peg.975   Thermoanaerobacter ethanolicus CCSD1
fig|1125975.3.peg.2106   Thermoanaerobacter indiensis BSB-33
fig|880478.3.peg.1806   Thermoanaerobacter siderophilus SR4
fig|399726.7.peg.1046   Thermoanaerobacter sp. X514

```
                                                                                                  nRyTTTAnYRYRnTAAArYr
fig|589861.3.peg.975   gtctatgcttgacatacattgaaaattaggatacaataaaagaagtacagtttattactttatcacgttacaaagacagagtaaattgtggggtgtttta   4.11
fig|1125975.3.peg.2106 gtctatgcttgacatacattgaaaattaggatacaataaaagaagtacagtttattactttatcacgttacaaagacagagtaaattgtggggtgtttta   4.11
fig|880478.3.peg.1806  gtctatgcttgacatacattgaaaattaggatacaataaaagaagtacagtttattactttatcacgttacaaagacagagtaaattgtggggtgtttta   4.33
fig|399726.7.peg.1046  gtctgtgcttgacatacattgaaaattaggatacaataaaagaaatacagtttattactttatcacgttacaaagacagagtaaattgtggggtgtttta
                       **** *************************************** *******************************************************

fig|589861.3.peg.975   ATGATATATTTGCCTGATGGTGTACAAGATTTTTTGCCGGAAGAATATAG
fig|880478.3.peg.1806  ATGATATATTTGCCTGATGGTGTACAAGATTTTTTGCCGGAAGAATATAG
fig|1125975.3.peg.2106 ATGATATATTTGCCTGATGGTGTACAAGATTTTTTGCCGGAAGAATATAG
fig|399726.7.peg.1046  ATGATATATTTGCCTGATGGTGTACAAGATTTTTTGCCAGAGGAATATAA
                       ************************************** ** *******
```

---

fig|1421016.3.peg.2602   Thermoanaerobacterium aotearoense SCUT27
fig|1094508.3.peg.1187   Thermoanaerobacterium saccharolyticum JW/SL-YS485
fig|858215.3.peg.1086   Thermoanaerobacterium xylanolyticum LX-11
fig|698948.3.peg.535   Thermoanaerobacterium thermosaccharolyticum M0795
fig|580331.4.peg.1866   Thermoanaerobacter italicus Ab9

```
                                                                                        nRyTTTAnYRYRnTAAArYr
fig|1421016.3.peg.2602 gaaaatgtacttgacacatagacaaaaataaattattatttaaataa--caagttattactttatcactttacaaagataaagtaa----agtaatagaa   4.08
fig|1094508.3.peg.1187 gaaaatgtacttgacacatagacaaaaataaattattatttaaataa--caagttattactttatcactttacaaagataaagtaa----agtaatagaa   4.08
fig|858215.3.peg.1086  gaaaatgtacttgacacatagacaaaaataaattattatttaaataa--caagtcattactttatcactttacaaaaataaagtaa----aatagtataa   5.02
fig|698948.3.peg.535   attaaagtacttgacacatggacaaaaataaattattatgtaaataa--caagtcattactttatcactctacaaagataaagtaatttagatggttagc
fig|580331.4.peg.1866  ttgtctgtgcttgacatacattgaaaattaggatacaataaaagaaatacagtttattactttatcacgttacaaagacagagtaa-----attgt----
                             ** ******* *     **** **   **  **  **  **  **  * *************  ****** * * *****      *  *

fig|1421016.3.peg.2602 gaggtgctttgATGAAGAATCTACCTGATGGAGTACAGGATTTTTTGCCTGATGAGTTGAGG
fig|1094508.3.peg.1187 gaggtgctttgATGAAGAATCTACCTGATGGAGTACAGGATTTTTTGCCTGATGAGTTGAGG
fig|858215.3.peg.1086  gaggtgctttgATGAAGAATTTACCTGACGGAGTACAGGATTTTTTGCCTGATGAGTTGAGA
fig|698948.3.peg.535   gaggtgctttgATGAAAAATTTACCTGATGGAGTACAGGATTTTTTGCCTGAGGAATTAAGA
fig|580331.4.peg.1866  ggggtgttttaATGATATATTTGCCTGATGGTGTACAAGATTTTTTGCCAGAGGAATATAAG
                       * **** *** ****   ** * ***** ** ***** *********** ** ** *  *
```

```
fig|520767.4.peg.2208       Thermovenabulum gondwanense strain R270
fig|632348.3.peg.1306       Caldicellulosiruptor kronotskyensis 2002
fig|521460.8.peg.1549       Anaerocellum thermophilum DSM 6725
fig|31899.10.peg.1973       Caldicellulosiruptor bescii strain DSMZ 6725

                                                                               nRyTTTAnYRYRnTAAArYr
fig|520767.4.peg.2208 tttcatatttttacttgccaaaattccacatataatttataatattatcaaattaactatttaaaatattaaagtaataaatttacataatggcggggt   4.06
fig|521460.8.peg.1549 ggctccactttttttgttgctttttttagttcttttgtaatataatatataa-catgatatctattcatcacttcattatgataaa-----gtaatgaaaggg-
fig|31899.10.peg.1973 ggctccactttttttgttgctttttttagttcttttgtaatataatatataa-catgatatctattcatcacttcattatgataaa-----gtaatgaaaggg-   4.69
fig|632348.3.peg.1306 ggctccactttttttgttgctttttttagttcttttgtaatataatatataa-catgatatctattcatcacttcattatgataaa-----gtaatgaaaggg-   4.69
                            * *****  ****     *    * * *    ******** * **   ** ***** *  *  * *   * *****      *****   ***

fig|520767.4.peg.2208 cgcataaaATGGGTAAAAAATTCACGGATTACTGCCCGCCGGGCAGTATT
fig|521460.8.peg.1549 -agaaaaaATGAGCAAAAAGTTTGTGGATTACTATCCTCCAGTGTCTGAT
fig|31899.10.peg.1973 -agaaaaaATGAGCAAAAAGTTTGTGGATTACTATCCTCCAGTGTCTGAT
fig|632348.3.peg.1306 -agaaaaaATGAGCAAAAAGTTTGTGGATTACTATCCTCCAGTGTCTGAT
                         * ****** * ***** **   ********  ** ** *    *  *
```

```
fig|525903.6.peg.232        Thermanaerovibrio acidaminovorans DSM 6589
fig|584708.3.peg.2236       Aminomonas paucivorans DSM 12260
fig|926567.3.peg.1615       Thermanaerovibrio velox DSM 12556

fig|525903.6.peg.232  aacacctccgttgattttccgcggcccccatgatatcatgcaaaccacgctttcggctaccaattcatcacgctaacaaggc-actggaggta----- 4.31
fig|584708.3.peg.2236 --cacctccgttgctgtcaaacggtttggatgatatcatgcggaccatgtcaagggatgaccctttatcccgatagcatgatgaacggagggaccgcc 3.93
fig|926567.3.peg.1615 aacacctccgttgcatttccgcggacaccatgataacatgggatccacgttttgggatgataatttatcacgctaatatgac-actggaggtaa---- 5.31
                        ***********   *    ***     ****** ****    *** *     ** *     ** *** ** **  * *   *  ***** *

fig|525903.6.peg.232  -tgaccatgtca--cctaaccgccaactcaggtgggtcccc----cgggag----------gaggtgccccgtaATGC--GGGTCCCC
fig|584708.3.peg.2236 atgaccgtcgcac-cgcagcgccccgcctccccgtcctcccgccgcgaaagcgcggaaccggaggtgggggccATGCTCCGAACCCC
fig|926567.3.peg.1615 -cagccatgagaaacgccgcacccaaatcgccggggaccccgtttgaagcg----------gaggtgcccggccATG---AGACCCCC
                          ** *   *  *    *  **         *    ***         *          ******    *  ***    *  ****
```

Legend and Abbreviations:

To validate candidate HisR-binding sites with scores below threshold (weak sites) we applied a phylogenetic footprinting approach to upstream regions of these genes using multiple sequence alignment of upstream gene regions for each group of closely-related orthologs as previosuly described in [1, 2]. Multiple alignments of DNA upstream regions were obtained by ClustalW2 integrated in the SEED platform. Genes/genomes with weak candidate HisR binding sites are highlighted in red in the header line, while other genes/genomes in black possess high-scored HisR sites. Predicted HisR-binding sites are highlighted by yellow boxes with a taxon-specific consensus shown on top. Gene coding regions are shown in capital red letters.

[1] Khoroshkin MS, Leyn SA, Van Sinderen D, Rodionov DA. 2016. Transcriptional regulation of carbohydrate utilization pathways in the *Bifidobacterium* genus. Front Microbiol 7:120.

[2] Ravcheev DA, Godzik A, Osterman AL, Rodionov DA. 2013. Polysaccharides utilization in human gut bacterium *Bacteroides thetaiotaomicron*: comparative genomics reconstruction of metabolic and regulatory networks. BMC Genomics 14:873.
