# Supplementary material for: Evolution of transcriptional regulation of histidine metabolism in Gram-positive bacteria
Source: BMC Genomics. 2022 Aug 25;23(Suppl 6):558. doi: 10.1186/s12864-022-08796-y (PMC9413887; doi:10.1186/s12864-022-08796-y)
Supplement: Supplementary file 6 — Additional file 6. Phylogenetic footprinting of upstream regions of genes controlled by candidate Histidine-dependent translational attenuators. [file 12864_2022_8796_MOESM6_ESM.pdf]

Additional File 6. Phylogenetic footprinting of upstream regions of genes controlled by candidate Histidine-dependent translational attenuators (see legend below).

A. Novel candidate His attenuator upstream of the *hisZGDBHAFIEJ* operon in the *Bacillus cereus* group (Bacillales).

222523.1.peg.1517 **Bacillus cereus ATCC 10987**
527024.3.peg.2634 **Bacillus thuringiensis serovar tochigiensis BGSC 4Y1**
1245029.3.peg.3591 **Bacillus anthracis str. Carbosap**
315730.11.peg.1914 **Bacillus weihenstephanensis KBAB4**
526997.3.peg.1686 **Bacillus mycoides DSM 2048**
315749.8.peg.1198 **Bacillus cytotoxicus NVH 391-98**

```
                     -35 box Promoter  -10 box                            Leader RBS      Leader start                                 His codons  Stop
315730.11.peg.1914  tatttacattcagaattaactgttatataatatgtaaaaaatcagaggagaaagggagtaagtattATGAATATCGTGCAATCTATTAAAATGCTTACAAAACTACATCATCATACATGA
526997.3.peg.1686   tatttacattcggaattaactgttatataatatgtaaaaaatcagaggagaaagggagtaagtattATGAATATCGTGCAATCTATTAAAATGCTTACAAAATTACATCATCATACATGA
527024.3.peg.2634   tatttacattcagaattaagtgttatatactatgtaaaaa-ttagtggagaaagggagtaagtattATGAAAACAATGCAATCTATTAAAATGCTTACACAACTACATCATCATACATAA
1245029.3.peg.3591  tatttacattcagaattatgtgttatatactatgtaaaaa-ttagaggagaaagggagtaagtattATGAAAACAATGCAATCTATTGAAATGCTTACACAACTACATCATCATACGTAA
222523.1.peg.1517   tatttacattcagaattaagtattatatactatgtaaaaa-ttagaggagaaagggagtaagtattATGAAAACAATGCAATCTATTAAAATGCTTACACAACTACATCATCATACATAA
315749.8.peg.1198   tatttactttctgaatagtttgttatataatgtgtgaaaa-taagcggaggaaaggagtaaggattATGTTTGCAATGAAGTGTATAAATATGCTTACAAAACTACACCATCATCCATAA
                    ******* *** ***  * ******* * *** **** * ** **** ** ******** ******       ** * * ***    ********* ** **** ****** * * *

                               Attenuator hairpin                                     terminator hairpin
315730.11.peg.1914  aacccttgaacctaacgtgaaa-acgtgtagggacaagggttattcccgttgtacctacactctactaaa-gagccttgtaggta--ttttgtctacaaggctctttt
526997.3.peg.1686   aacccttgaacctaacgtgaaa-acgtgtaggcacaagggttattcccgttgtacctacactctactaaa-gagccttgtaggta--ttttgtctacaaggctctttt
527024.3.peg.2634   aacccttgaacctagcgtgaaa-acgagtaggtgcaagggttattcccgttgtacctactcgctactaaa-gagccttgtaggtatattttatctacaaggctctttt
1245029.3.peg.3591  agcccttgaacctaacgtgaaa-acgtgtaggtgcaagggttattcccgttgtacctacacgctacgaaa-gagccttgtaggtatattttatctacaaggctctttt
222523.1.peg.1517   agcccttgaacctagcgtgaaa-acgtgtaggtgcaagggttattcccgttgtacctacacgctactaaa-gagccttgtaggtatattttatctacaaggctctttt
315749.8.peg.1198   aacccttgctcctagcgtgaaaaacgtgtaggtacaagggttattcctgttgtacctacactctataaaaagagccttgtaggta--ttttttctacaaaggctcttt
                    * ******  **** ******** ** *****  ************* *********** * ***   ** **************  **** ******* *    ***

                                                                       hisZ gene start
315730.11.peg.1914  tattttcggtttgggtggatggaatgg-cataaattaaagagggaagggtgtgtgaattcagATGACAAAGTGGAAACGGGCAAATCCGAATGGAACGAG
526997.3.peg.1686   tattttcggtttgggtggatggaatgggcataaattaaagagggaagggtgtgtgaattcagATGACAAAGTGGAAACGGGCAAATCCGAATGGAACGAG
527024.3.peg.2634   tattttcggtttaggtgcatgaaataagcatacattagaaagggaaaggtgtgagaatttagATGACAAAATGGAAACGGGCAAACCCAAATGGAACGAG
1245029.3.peg.3591  tattttcggtttaggtgcatgaaataagcatacattagaaagggaaaggtgtgagaatttagATGACAAAATGGAAACGGGCAAACCCAAATGGAACGAG
222523.1.peg.1517   tattttcggtttaggtgcatgaaataagcatacattagaaagggaaaggtgtgagaatttcgATGACAAAATGGAAACGGGCAAATCCAAATGGAACGAG
315749.8.peg.1198   ttcttttgtttgtgattgttatggtgagcatatacataaaaagaaaggg----ggagtttacATGACGAAGTGGAGACGTACAAAACCAAATGGAACGAG
                    *  *** *  *  *     *    *    **** *    *    * ** **      * **   ***** ** **** ***   **** ** ***********
```

B. Novel candidate His attenuator upstream of the *hisZGCBHAFIE* operon in the Peptostreptococcaceae group (Eubacteriales).

1496.1.peg.3371 Clostridioides difficile 630

445973.7.peg.1529 Intestinibacter bartlettii DSM 16795

```
                   Leader RBS                 Leader start                          His codons                    Stop
1496.1.peg.3371   --aattatttgggaggaaattatcatg-GTGAGATTAAATATAGTAAAATTAAATACAAAATTAAATCATCACCATCATCATAGATTAAAGGATTGTAAt-atgaaattaat
445973.7.peg.1529 ggaggaatggaagATGAATTTAATATACACACAATTTAATACAATAACAATAGA---ACAATTAAATCATCATCACCATCATAGATCTTAGggattgtaattatgaaatttat
                      *   **  ** *** ***  **       *** **** * *** * ** *   * ************* ** **********  ************ ******** **

                                 Attenuator hairpin                                           Terminator hairpin
1496.1.peg.3371   tcatagacacaagccctgtttttagtgtaattggtcttgtatctatgatgggaagtaattttaagagaaaattatagccatataggtataaagcctatgtggc
445973.7.peg.1529 tcatagatgcaagccctatttgtgttagctaaaggcttgagtctatggtgggatagaaattactaaaccatgtaggtaaaagcctgcatggttttttaatac
                  *******  ******** *** *  *       * ****  ****** ***** ** ****           **

                                                                                                   hisZ gene start
1496.1.peg.3371   ttt--------tttattgttttaaa-------------tttgcaaatataaga-gtaaatttaatg------aagagggtgagaat-------GTGAAATTTT
445973.7.peg.1529 tattttaatactatatagttttagagcatataaaatattatttaaaaattagatataagtttaatataagacagaaaggtatgagtgagtataATGAAATTTT
                      *   *  * *** ****** *             * *  *** ** ***  *** ******       *  * ***  ** *        *********
```

1122184.3.peg.1024 Lutispora thermophila DSM 19022

```
tatactacaatagcatactaagtataataattatggattttagttacagaaaggaagtcaactATGTTTACTGTATTTATATTGAATTCTAATAAGAAGCATCATCACCATCATAGTGAC
AGGGTTTGTATTCTAGGCATATAAttatgcataggtacaatccctgattctgttttgaatttggggttgtacctatgatggttaagatataaaaaaaagaagccatataggtatacctа
tatggcttcttttttaaaaaaccataggaaagtataattataccttaatatagactttttgtaatgggtttcaagaaaggcttccttgttattaatatattttaaatagaagcctttct
tatgcagcaattaatggcaggaggcaggaaaagtaaATGCAATTT
```

1121315.3.peg.2331 Terrisporobacter glycolicus ATCC 14880

```
ATGATGTATATTATAAATGAAAAACTTAATAGCAAATTAAATACAACATTATATTATCATCATCATAGATCCAAGGGATTGTAAtcatgaaattttttcatagatacaagccctattttaagtacagcaaatg
ggcttgtatctatgatggtatatgaaacttataaaccatataggcatagcctatatggtttttttaattactagaatattattttgtctgtggaaaacttctagattaaagtattatcaatacatttaagaa
gtagtaaaaaagtgaattttgagcaacggagttgcctgaagcggtagcaaaatgattcgccaacatgttgctcaaaggcatctcgttggcgacataaagtgtagcgcccacgcagtggcttaagcgaagcga
attttaataagtaagaaaggactacaaggaggaaatATGAAGTATT
```

```
L.thermophila TAAttatg--------cataggtacaatccctgattctgttttgaatttggggttgtacctatgatggttaagatataaaaaaaagaagccatataggtata-cctatatggcttcttttttt
T.glycolycus  TAAtcatgaaattttttcatagatacaagccctattttaagtacagcaaatgggcttgtatctatgatggtatatgaa----acttataaaccatataggcatagcctatatggtttttttaat
              **** ***        ***** ***** ****  **    *        **** ***** **********          *  * ** ********* *** ********* ** ***  *
```

499177.3.peg.513 Acetoanaerobium sticklandii DSM 519

```
atctaaggagagatactaATGAACCTTCTAATACTTACAACTGTACAACTACATCATCATTATTAGaagggtatgagccgttattggtccagccctgttcgtgttttgaaagggctaacttctaataatggc
aaaaataagatatattttatttttagtacgcacaagagccattttggatttatagtccaaggtggctttttttatttcaataaaaaggagagatattATGCTAAGAATAGCACTTGCAAAAGGAAGGCTTCA
```

C. Novel candidate His attenuator upstream of the *hisZGDCBHAF(IE)* operon in the Erysipelotrichales group.

451640.5.peg.1377 Catenibacterium mitsuokai DSM 15897
1297794.4.peg.3389 Coprobacillus cateniformis JCM 10604
999415.3.peg.1341 Eggerthia catenaformis OT 569 = DSM 20559
445974.6.peg.1707 Erysipelatoclostridiumramosum DSM 1402
69824.3.peg.1410 [Clostridium] cocleatum strain DSM 1551
428126.7.peg.165 [Clostridium] spiroforme DSM 1552

```
                           leader RBS      leader start                His codons             Stop     attenuator hairpin
445974.6.peg.1707 tacaatgaga-ggtgttaattATGAAAACAACAACAAATTTACAATTA-CATCACCATCATTGGATT---GCCTAGggttttatc--agtttattgatacaaccc
69824.3.peg.1410  tacaatgaga-ggtgctaattATGAAAACAATCACAAATTTACAACTA-CATCACCATCATTGGATT---GCCTAGggttttatc--agttt-ttgatacaaccc
428126.7.peg.165  tacaatgaga-ggtggataatATGAAAAAACTAATTAATTTACAATTA-CATCACCATCATTGGATT---GCCTAGggttctatc--aacgt--tgatacaaccc
1297794.4.peg.3389-aaaatgaggtgatatttATGAAAAACACAATTTTATATTCAAAATTT-CATCACCATCATTGGAAC---AACTAGggttatatctaaaagaatagatacaaccc
999415.3.peg.1341 aaaaatgaggtgattact-ATGCAAAACTATACAGTTA--AACAATTT-CATCATCACCATTGGCGT---ATTTAGggttatatc---attt---gatacagccc
451640.5.peg.1377 aaaactgaggtgattatttATGAAAAATACAATTTTTGTCAACAATTTACACCATCACCATCGTGGTTCTAGTTAGggttatatc---aatta--gatacagccc
                   * * ****  * *          **               * ** *  ** ** ** *** *          ******* ****          ****** ***

                                                                       Terminator hairpin                                   hisZ gene start
445974.6.peg.1707 attttg-tgttaaatattatttgggtgtatctaatgac---aatattactttag-cgagtcattagtgtaatgactcgtttttttgtaactaaa-ggggaaataaaATGGAAGAA
69824.3.peg.1410  attttg-tattaaatatagtttgggtgtatctaatgac---aatattactttag-cgagtcattagtgtaatgactcgcttttttataactaaaaggggaaaagaaATGGAAGAA
428126.7.peg.165  attttg-tgtataatatt-tatgggtgtatctaatggc---aatttgtttataaacgagtcattagta-aatgactcgttttttt---aatagaaggggaatttagGTGGAAGAA
1297794.4.peg.3389attttg-tgtat-ataaaatttggttgtatctgatggcttagaggtttatatgg-cgagtcattaa---gactcgtcattttt-ataagagaggggggataaaaaATGGAAGAA
999415.3.peg.1341 gttttgttttacaattaagcaaggctgtca--atctaat----aacactagaaacgaggttattag-----tacctcgtttttt-attatagaggaggaaaaga--ATGGATGAA
451640.5.peg.1377 gttttgacatacaactcaaaaaaacggacacgaaacaat----gacataagggaacaggtcattg------gacctgttcctttttatatataaggggacatgactTTGGAAGAA
                   *****   *  **            *                               ** ***                 ***    *       *           *** ***
```

Legend and Abbreviations:

To identify candidate His attenuators we applied a phylogenetic footprinting approach to upstream regions of *his* genes using multiple sequence alignment of upstream gene regions for each group of closely-related orthologs. Multiple alignments of DNA upstream regions were obtained by ClustalW2 integrated in the SEED platform. Legend for attenuator elements:

LEADER PEPTIDE His codons Stop codon attenuator hairpin terminator hairpin Potential antiterminator Coding region of downstream gene
