# Supplementary material for: Evolution of transcriptional regulation of histidine metabolism in Gram-positive bacteria
Source: BMC Genomics. 2022 Aug 25;23(Suppl 6):558. doi: 10.1186/s12864-022-08796-y (PMC9413887; doi:10.1186/s12864-022-08796-y)

Additional file 1. Maximum-likelihood phylogenetic tree for the HisR proteins in studied genomes.

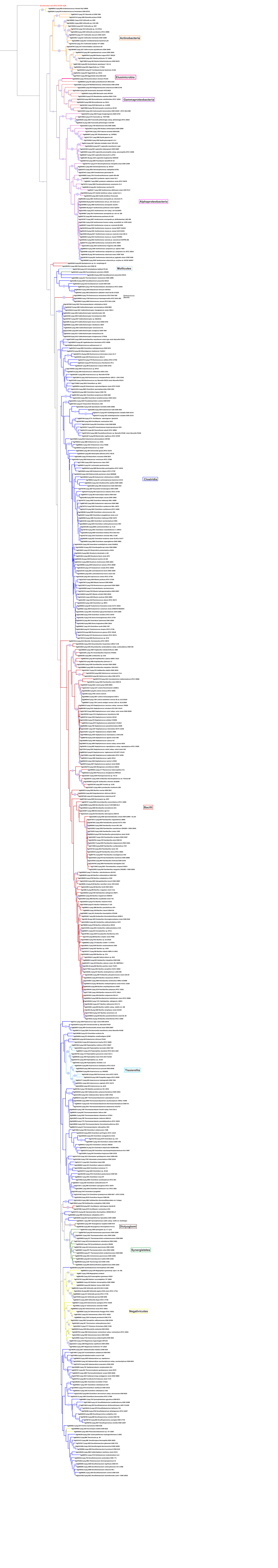

Supplement: Supplementary file 1 — Additional file 1. Maximum-likelihood phylogenetic tree for the HisR proteins in studied genomes. [file 12864_2022_8796_MOESM1_ESM.pdf]
